# Manipulating root-associated microbiomes to boost drought resistance in dryland winter wheat with *Streptomyces pactum* Act12

**DOI:** 10.1186/s12866-026-04812-3

**Published:** 2026-02-10

**Authors:** Meng Li, Rennan Yang, Qiannan Bai, Zhenping Yang, Tingmiao Huang, Yuejing Qiao, Bin Yang, Jie Chen, Wen Lin

**Affiliations:** 1 College of Agriculture, Shanxi Agricultural University, Taigu, 030801 China; 2 Shanxi Provincial Key Laboratory of Crop Ecology and Efficient Water & Nutrient Use, Shanxi Agricultural University, Taigu, 030801 China; 3 Institute of Wheat Research, Shanxi Agricultural University, Linfen, 041000 China

**Keywords:** Drought stress, Wheat yield, Rhizosphere microbiome, Rhizoplane microbiome, Microbial function

## Abstract

**Background:**

Drought critically compromises agricultural productivity and threatens sustainable wheat production. *Streptomyces pactum* Act12 confers benefits to plant growth under drought stress, but its possible effects on root-associated microbiomes remain understudied. Here, shotgun metagenome sequencing and culture-dependent approaches were integrated to investigate the responses of rhizosphere and rhizoplane microbiomes in dryland winter wheat to exogenous *S. pactum* Act12 and their potential linkage to plant drought resistance.

**Results:**

Seed biopriming with *S. pactum* Act12 increased plant aboveground dry weight at flowering (by 63.2%) and maturation (by 41.9%) stages, leading to improved grain yield (by 8.7%). Microbial inoculation reduced malondialdehyde contents in wheat leaves and roots at the flowering stage alongside compartment-specific alterations in soil microbiomes. Metagenomic analysis revealed inoculation-induced enrichment of distinct taxa in rhizosphere soils (flowering: Fibrobacterota, *Altererythrobacter*; maturation: Mucoromycota, *Rhodospirillum*) and rhizoplane soils (flowering: Pseudomonadota, *Serratia*; maturation: Candidatus_Pacebacteria, *Variovorax*). Functional profiling showed up-regulation of key pathways related to oxidative phosphorylation in inoculated rhizosphere soils at the flowering stage. In rhizoplane soils, ABC transporters and pyrimidine metabolism were up-regulated across stages upon inoculation. Two key strains isolated from rhizoplane soils, designated *Glycomyces lechevalierae* A4 and *Microbacterium algeriense* B3, demonstrated the ability to enhance drought resistance in wheat seedlings.

**Conclusions:**

Inoculation of *S. pactum* Act12 heightens drought resistance in dryland winter wheat through compartment-specific phylogenetic restructuring and functional reprogramming of root-associated microbiomes.

**Supplementary Information:**

The online version contains supplementary material available at 10.1186/s12866-026-04812-3.

## Introduction

Drought is a natural hazard marked by pronounced regional disparities, with heavy consequences for agroecosystems in arid and semi-arid areas [1]. As an abiotic stressor to agricultural productivity, drought causes up to 70% yield reduction in wheat crops [2]. In the wake of global warming, drought is projected to intensify and affect 60% of the global wheat-growing area by 2100 [3]. Therefore, developing effective strategies to enhance crop drought resistance is crucial for boosting wheat production and safeguarding global food security. Wheat (*Triticum* spp.) is the third major cereal crop after rice and maize, contributing 21% of global protein and caloric intake and sustaining over 35% of the world’s population [4–6]. Crop yield improvement is a top priority for wheat production as wheat consumption continues to increase with a growing population and dwindling arable land [7, 8].

The introduction of exogenous functional microorganisms can stimulate plant drought resistance and sustain high crop yields. These plant beneficial effects are attributed to multi-level mechanisms, including secretion of microbial phytohormones stimulating root growth and induction of plant osmoprotectant biosynthesis for cellular osmotic balance. Exogenous microorganisms additionally play a role in the up-regulation of drought-responsive plant genes and the restructuring of soil microbiomes [9, 10]. Compared to conventional agricultural practices, microbial inoculation offers a range of ecological and economic advantages, such as environmentally benign and cost-effective procedures [11]. The role of microbial inoculation in enhancing plant drought resistance has been reported in wheat [12, 13], maize [14], rice [15], and oat [16]. In particular, *Streptomyces* spp. are promising microbial agents that confer drought resistance in crops. Many *Streptomyces* strains exhibit disease tolerance, plant growth-promoting traits, and spore persistence in arid soils [17, 18]. Accumulating evidence indicates that *Streptomyces* can improve wheat growth performance under drought stress by regulating plant growth, physiology, gene expression, and metabolism [13, 19, 20]. However, the responses of root-associated microbiomes to exogenous *Streptomyces* in wheat remain understudied, hindering our understanding of plant-soil-microbe interactions.

Soil microorganisms in the root zone establish close associations with host plant performance. Some taxa contribute to plant drought resistance by improving soil water availability, promoting root development, and enhancing plant antioxidant capacity [21, 22]. Root-associated microbiomes are spatially compartmentalized into the rhizosphere and rhizoplane [23], each representing distinct ecological niches with divergent functional contributions to plant development [24]. Hence, disentangling the dynamics of rhizosphere and rhizoplane microbiomes is essential to elucidate their role in plant drought resistance. Previous studies have shown that exogenous functional microorganisms mediate the enhancement of crop drought resistance by shaping soil microbial communities [25, 26]. However, root-associated microbiomes were mainly investigated as a single entity [22, 27], lacking systematical analysis distinguishing between rhizosphere and rhizoplane microbiomes in *Streptomyces*-inoculated wheat.

High-throughput sequencing-based metagenomics and conventional culture-dependent techniques (isolation and cultivation) are complementary microbiological methods. Although high-throughput sequencing delivers near-exhaustive taxonomic profiles of soil microbiomes, this method precludes targeted strain isolation and phenotypic validation [28, 29]. Culture-dependent techniques allow functional characterization of viable isolates. However, less than 1% of extant soil microbiomes can be recovered under culture conditions, limiting comprehensive assessment [30, 31]. To overcome the limitations of single methods, high-throughput sequencing and isolation-cultivation techniques should be integrated, enabling a holistic characterization of soil microbiomes.

*Streptomyces pactum* Act12 is a functional microorganism previously shown to enhance drought resistance in wheat [13, 20]. In this study, a dryland field experiment was conducted to characterize rhizosphere and rhizoplane microbiomes in response to exogenous *S. pactum* Act12 and explore their association with drought resistance in winter wheat. The aim of this study was to elucidate how root-associated microbiomes contribute to *Streptomyces*-mediated drought resistance in dryland winter wheat. Results of this study have wide implications for the use of *S. pactum* Act12 in sustainable wheat production in arid and semi-arid areas. To our knowledge, this study is the first to resolve the rhizosphere- and rhizoplane-specific responses of root-associated microbiomes to *Streptomyces* in dryland plants by integrating shotgun metagenome sequencing with culture-dependent approaches.

## Materials and methods

### Experimental site and materials

The study took place at the Wheat Experimental Base of Shanxi Agricultural University in Yuncheng, Shanxi Province, China (35°24’1"N, 111°25’18"E). The experimental site is located in a typical semi-arid region under continuous cropping of wheat. Local precipitation and temperature records during the experimental period are presented in Fig. S1. Before the commencement of the experiment, the surface soil (0–20 cm) had a pH value of 7.04 and contained organic matter of 12.97 g kg⁻¹, alkali-hydrolyzable nitrogen of 55.55 mg kg⁻¹, available phosphorus of 5.70 mg kg⁻¹, and available potassium of 90.00 mg kg⁻¹ on an air-dry weight basis.

*Streptomyces pactum* Act12 was isolated from an extremely arid soil of alpine meadow on the Qinghai-Tibet Plateau [32]. The test strain was provided by the Laboratory of Microbial Resources, College of Resources and Environment, Northwest A&F University (Yangling, Shaanxi Province, China). A powdered form of live spore preparation was produced through solid state fermentation by Boqin Bio-Engineering Co., Ltd. (Yangling, Shaanxi Province, China), with a viable spore count of 1 × 10^8^ colony-forming units g^–1^. The winter wheat (*Triticum aestivum* L.) variety used in the experiment was Linhan 8, bred by the Institute of Wheat Research at Shanxi Agricultural University (Linfen, Shanxi Province, China).

### Experimental design and treatments

The field experiment was carried out between October 8, 2023 and May 31, 2024. Two treatments were established: microbial inoculation and no inoculation (control). Each treatment was replicated in three 5 × 10 m plots arranged in a completely randomized design. Wheat seeds were moistened with 4% (*w*/*w*) carboxymethyl cellulose sodium (6.0 g L⁻¹) and then dusted with 6% (*w*/*w*) spore powder of *S. pactum* Act12. Autoclave-sterilized soil instead of spore powder was used as the control. On October 8, 2023, wheat seeds were drilled at a rate of 210 kg ha^–1^ with a row spacing of 34 cm. Before sowing, compound fertilizer (N-P₂O₅-K₂O = 21-17-6) was applied at 900 kg ha⁻¹ and incorporated into the soil by rotary tillage. Routine field management was maintained, and no additional fertilizer or irrigation was applied after sowing until grain maturation.

### Plant performance measurements

In each plot, 10 plants were randomly selected at both flowering (May 2, 2024) and maturation (May 31, 2024). The aboveground plant parts were oven-dried at 105 °C for 1 h and then at 75 °C until constant weight. Aboveground dry weight was determined using a digital balance (0.01 g precision). At the maturation stage, two 1-m-long rows per plot were randomly selected and manually harvested to determine spike numbers, and the grain yield was reported at 13% moisture. Then, 20 representative spikes were sampled to count total grains, calculate the number of grains per spike, and determine thousand-kernel weight after air-drying. Additionally, a composite sample was formed by mixing 30 random plants from each plot at the flowering stage. The malondialdehyde (MDA) contents in flag leaves and 0–20 cm roots were quantified by thiobarbituric acid assay [33].

### Soil sampling

At the flowering and maturation stages, three 40-cm rows per plot were selected and diagonally sampled to collect 0–20 cm soil cores. After gentle shaking to remove loosely adhering bulk soil, the 0–5 mm adhering soil (i.e., rhizosphere soil) was brushed off and passed through a 5-mm sieve. Rhizosphere soil samples from each plot were pooled as one replicate and divided into two parts; one part was stored at − 80 °C for metagenomic analysis, and the other part was used for culture-dependent analysis. Thereafter, the roots of 50 plants from each plot without rhizosphere soil were randomly selected and weighed fresh (M1). The roots were ultrasonicated at 40 kHz in sterile water (1 min) to remove the rhizoplane soil and then weighed again (M2). The difference in root weight (M1–M2) was calculated to represent the rhizoplane soil dry weight. The rhizoplane soil suspension was divided into two portions; one portion was centrifuged (4000 r min^–1^, 10 min) and the soil precipitate was collected and stored at − 80 °C until metagenomic analysis, with the other portion used for culture-dependent analysis.

### Shotgun metagenome sequencing

A total of 24 soil samples (two treatment × two compartments × two stages × three replicates) were used for sequencing analysis. DNA extraction was performed using the QIAamp^®^ Fast DNA Stool Mini Kit (Qiagen, Hilden, Germany). After quantification and quality assessment, the extracted DNA was amplified on an ABI 2720 thermocycler (Applied Biosystems, Foster City, CA, USA). Purified PCR products were processed into libraries using the TruSeq Nano DNA LT Sample Preparation Kit (Illumina, San Diego, CA, USA). High-throughput sequencing was conducted on an Illumina NovaSeq 6000 platform (Illumina) by OE Biotech Co., Ltd. (Shanghai, China). Raw reads in the FastQ file were trimmed and filtered using fastp v0.20.1 [34]. Sequence alignment against the host genome was performed to remove genomic contaminants using BBmap v38.93-0 [35]. Metagenomic assemblies were generated with MEGAHIT v1.2.9 [36]. Open reading frames within scaffolds were predicted using Prodigal v2.6.3 and translated into amino acid sequences [37]. MMSeqs2 v13.45111 was used to construct a non-redundant gene catalog, with clustering parameters set to 95% sequence identity and 90% coverage [38]. The longest gene was selected as the representative sequence of each gene set. Clean reads of each sample were aligned against the non-redundant gene set (95% identity) using salmon v1.8.0, and gene-level transcripts per million values were calculated [39]. The taxonomy of species was obtained as a corresponding taxonomy database of the NR Library (https://www.ncbi.nlm.nih.gov/), and the abundance of species was calculated using the corresponding abundance of genes. Alpha-diversity metrics (Chao1, ACE, Shannon, and Simpson indices) were estimated at the specie level. Functional annotation was performed by aligning representative sequences from the non-redundant gene catalog against the Kyoto Encyclopedia of Genes and Genomes (KEGG) pathway database (http://www.kegg.jp/ or http://www.genome.jp/kegg/) using DIAMOND v2.1.3 [40].

### Enumeration, isolation, and identification of soil microorganisms

Culturable actinobacteria (A), bacteria (B), and fungi (F) in rhizosphere and rhizoplane soils were enumerated and isolated on respective media using serial dilution and spread plate techniques [41]. The total viable count (A+B+F), A/F ratio, and B/F ratio were calculated for each sample. The dominant strains (representing ≥ 5% of respective microbial community) in each treatment were purified and sent to Personalbio Technology Co., Ltd. (Shanghai, China) for molecular characterization. After DNA extraction, PCR amplification was performed using the primers 27 F/1492R (forward: 5’-AGAGTTTGATCCTGGCTCAG-3’; reverse: 5’-GGTTACCTTGTTACGACTT-3’) for actinobacteria and bacteria [42], and ITS1/ITS4 (forward: 5’-TCCGTAGGTGAACCTGCGG-3’; reverse: 5’-TCCTCCGCTTATTGATATGC-3’) for fungi [43]. Qualified PCR products were recovered, purified, and sequenced on an ABI 3730-XL DNA analyzer (Applied Biosystems). Contiguous sequences of the dominant strains were aligned with the NCBI database (https://blast.ncbi.nlm.nih.gov/Blast.cgi). Taxonomic identities were assigned based on the closest matched sequences.

### Functional validation of key strains

*Glycomyces lechevalierae* (A4) and *Microbacterium algeriense* (B3) were screened out from the dominant strains as key strains. Their genus-level shifts in metagenomic profiles matched the quantitative responses in enrichment cultures to exogenous *S. pactum* Act12. Both strains were grown in selective media to validate their potential functions in drought resistance. The tested functions were nitrogen fixation, phosphorus and potassium solubilization [44, 45], and production of 1-aminocyclopropane-1-carboxylate (ACC) deaminase, siderophores, IAA [46], and extracellular polysaccharides [47]. DIAMOND v2.1.3 was used to quantify functional genes related to nitrogen fixation (*glnA*, *asnB*) [48], potassium solubilization (*mdh*, *ackA*, *trkH*) [49, 50], and IAA biosynthesis (*mtrB*, *iaaB*, *trpB*) [51] in *Glycomyces* and *Microbacterium*.

The effects of inoculation with the key strains on drought resistance in winter wheat were validated by a seed germination assay based on the method of Qiao et al. [52] with minor modification. Seeds of uniform sizes were surface disinfected with a 5% (*w*/*v*) sodium hypochlorite solution for 30 s followed by 70% (*v*/*v*) ethanol for 30 s, and then rinsed with sterilized water three times. Drought stress was simulated by adding a 15% (*w*/*v*) polyethylene glycol (PEG)−6000 solution to seeds in petri dishes. Based on a preliminary experiment, cell-free culture broths of *G. lechevalierae* A4 (10-fold diluted) and *M. algeriense* B3 (10^6^-fold diluted) were applied. The control groups received an equal volume of sterilized water with PEG solution (drought control) and without PEG solution (normal control). Each treatment was replicated in three petri dishes. Germinated seeds in each Petri dish were counted, and germination rates were calculated at 12-h intervals. Six uniformly growing seedlings were randomly selected from each dish at 96 h to record fresh weight, radicle length, and hypocotyl length.

### Data analysis

R v4.1.2 (R Core Team, R Foundation for Statistical Computing, Vienna, Austria) was used to assess the differences in alpha-diversity metrics and taxa (pathway, gene) relative abundances between groups. Culturable microbial numbers and plant growth parameters were compared using *t*-tests. Duncan’s multiple range tests were performed for multiple comparisons of seed germination rate and seedling growth parameters among treatments. Both tests were carried out using IBM SPSS v26.0 (IBM Corp., Armonk, NY, USA), with statistical significance determined by *P* < 0.05. Multidimensional data visualization was achieved using Origin v2024 (OriginLab Corp., Northampton, MA, USA) for plant parameters, root-associated microbiome structure (top 10 most abundant genera), functional gene abundances, and culturable community composition. Beta-diversity analysis was conducted using principal coordinates analysis (PCoA) based on the Bray-Curtis distance algorithm in R v4.1.2 to elucidate microbiome variation among samples. Permutational multivariate analysis of variance (PERMANOVA) was performed with the adonis function in R *vegan* package (999 permutations, *P* < 0.001) to assess the explanatory power of experimental factors for microbiome dissimilarities. The abundance profiles of differentially abundant taxa (top 10 phyla and genera) and predicted functional capacities (KEGG Level 3) were identified with false discovery rate (FDR) correction, and their abundance profiles were visualized using the R *patchwork* and *ggplot2* packages. Mantel tests were conducted using the ChiPlot online platform (https://www.chiplot.online/) to examine the effects of root-associated microbiomes (structure and function) and culturable community composition (total number and population ratios) on plant performance.

## Results

### Diversity of root-associated microbiomes

#### Compartment-specific, core–pan gene signatures

Sequencing results showed that microbial inoculation altered gene cluster abundance in both rhizosphere and rhizoplane soils across plant growth stages (Fig. S2a and b). Inoculated rhizosphere soils yielded more unique gene clusters than the control group across stages (flowering: 60,053 vs. 57,895; maturation: 112,385 vs. 67,825). Inoculated rhizoplane soils contained fewer gene clusters than the control group at the flowering stage (95,647 vs. 98,645), whereas the opposite pattern was observed at the maturation stage (85,538 vs. 60,239). The converging core-pan gene curves confirmed adequate sequencing depth for all samples (Fig. S2c and d).

#### Alpha- and beta-diversity patterns

Rhizosphere soils showed significantly lower microbial diversity in terms of Chao1, ACE, Shannon, and Simpson indices than rhizoplane soils across plant growth stages (Fig. S3). At the flowering stage, microbial inoculation reduced the Shannon (by 6.4%) and Simpson indices in rhizoplane soils compared to the control group (*P* < 0.05). At the maturation stage, microbial inoculation increased the Chao1 (by 4.3%) and ACE (by 4.4%) indices in rhizosphere soils, despite reducing the Simpson index (*P* < 0.05). Additionally, microbial inoculation increased the Shannon and Simpson indices in rhizoplane soils (*P* < 0.05).

**Fig. 1:**
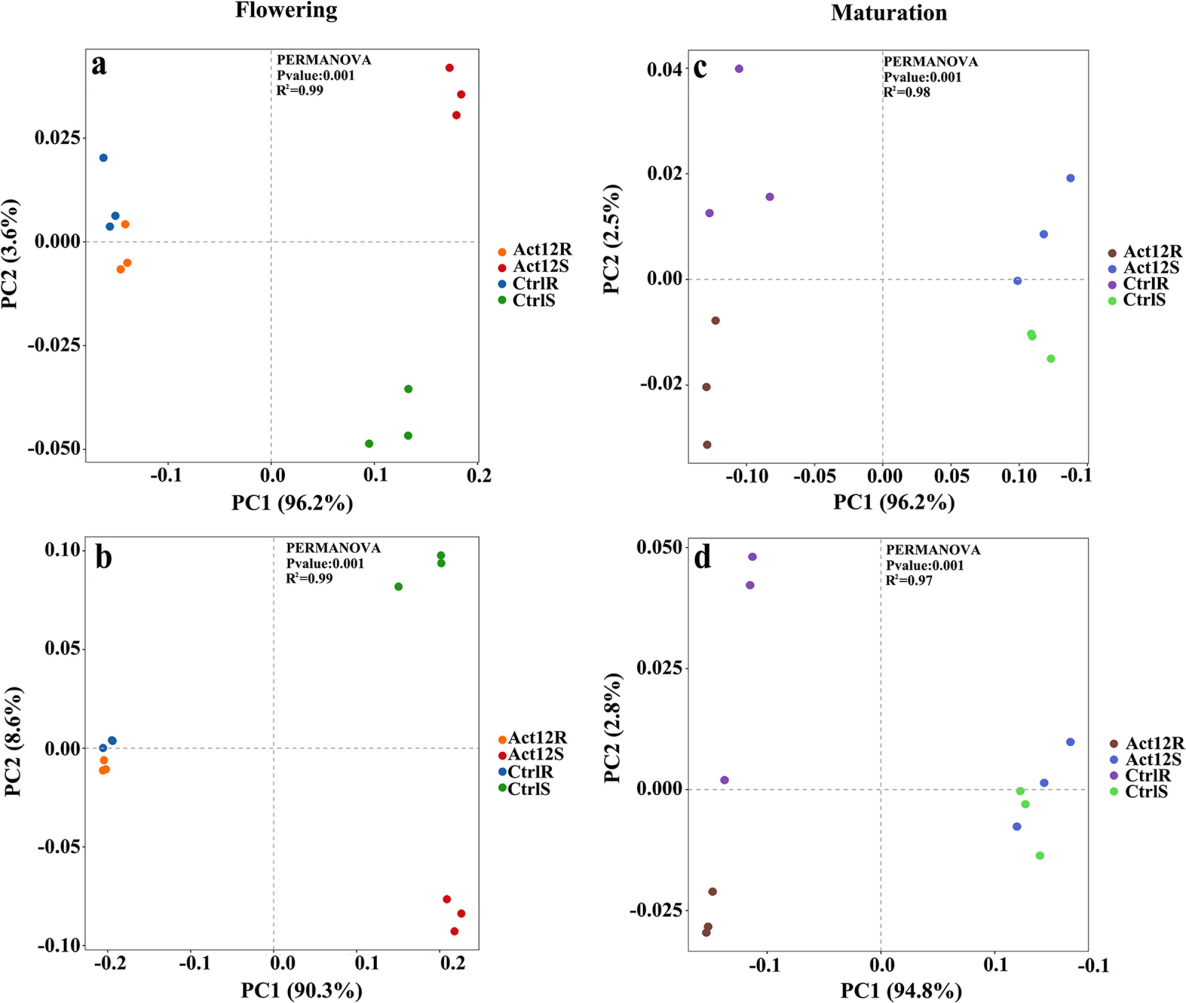
Principal coordinates analysis of soil microbial community. **a**, **c** phylum level; **b**, **d** genus level. Act12R, rhizosphere of *Streptomyces pactum* Act12-inoculated wheat; Act12S, rhizoplane of *S. pactum* Act12-inoculated wheat; CtrlR, rhizosphere of non-inoculated wheat; CtrlS, rhizoplane of non-inoculated wheat

In the PCoA plots, rhizosphere and rhizoplane samples from each stage were distributed across different quadrants (Fig. 1), indicating substantial variation in microbiome composition between compartments at both the phylum and genus levels. At the flowering stage, rhizosphere samples overlapped, whereas inoculated and control rhizoplane samples were located in distinct quadrants (Fig. 1a and b). This pattern was reversed at the maturation stage, when inoculated and control rhizosphere samples showed a clear separation, with rhizoplane samples in close proximity (Fig. 1c and d). These results indicate that microbial inoculation exhibited a stronger modulatory effect on the diversity of rhizoplane microbiomes in the middle growth stage of wheat, and rhizosphere microbiomes became more responsive in the late stage.

### Structure and function of root-associated microbiomes

#### Dominant and differential taxa abundance

**Fig. 2:**
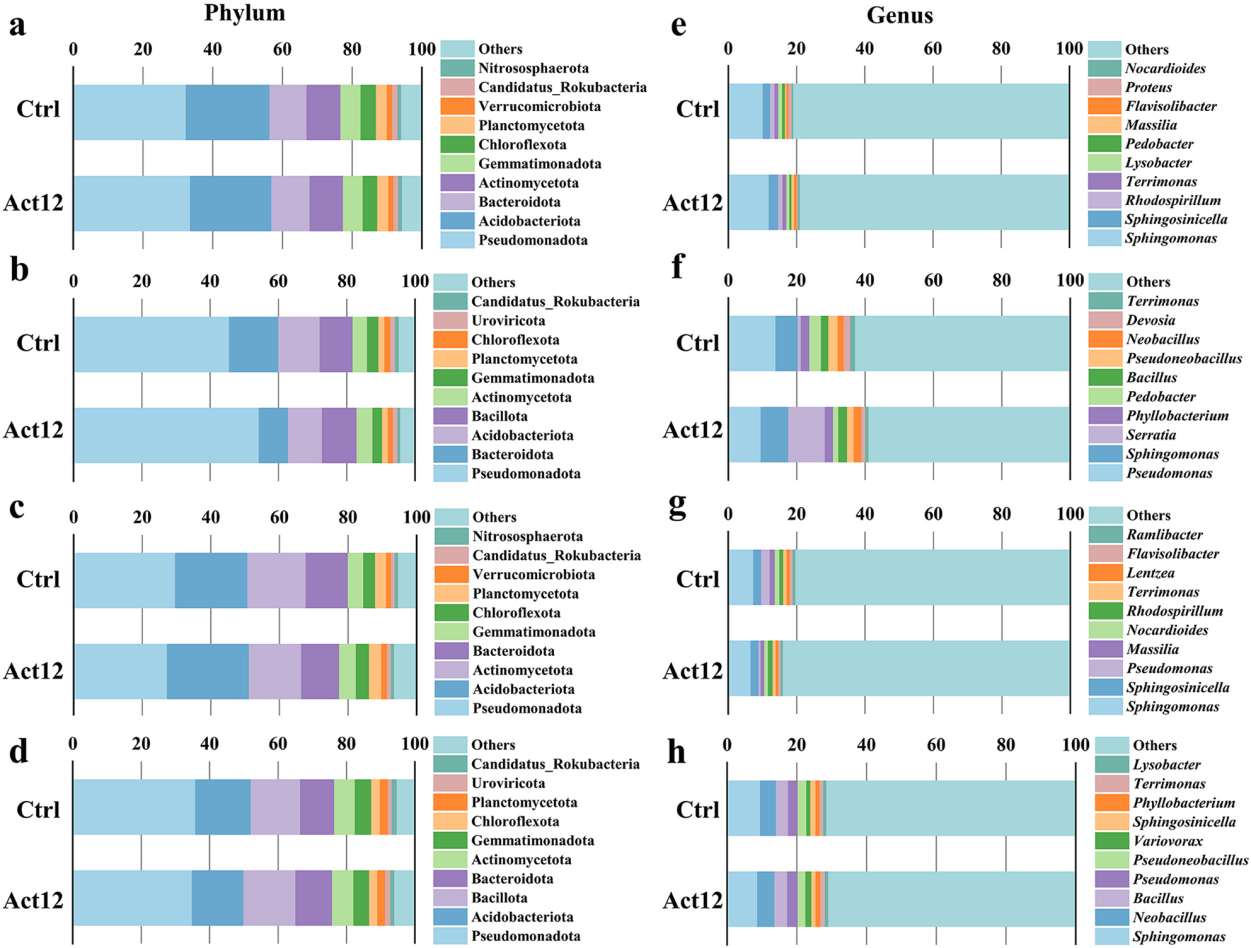
Relative abundance of the top 10 taxa. **a**, **e** rhizosphere at flowering; **b**, **f** rhizoplane at flowering; **c**, **g** rhizosphere at maturation; **d**, **h** rhizoplane at maturation

At the phylum level, Pseudomonadota, Acidobacteriota, and Bacteroidota were the most abundant taxa in rhizosphere soils across different groups during the flowering stage (Fig. 2a). Rhizoplane soils of the control group were dominated by Pseudomonadota (45.6%), Bacteroidota (14.4%), and Acidobacteriota (12.1%), whereas Bacillota emerged as the second-ranked phylum after inoculation (10.2%) (Fig. 2b). During the maturation stage, the primary phyla in rhizosphere soils of different groups were Pseudomonadota, Acidobacteriota, and Actinomycetota (Fig. 2c). In particular, rhizoplane soils of the control group mainly harbored Pseudomonadota (35.9%), followed by Acidobacteriota (16.2%) and Bacillota (14.4%); Bacillota (15.1%) surpassed Acidobacteriota (15.1%) in rhizoplane soils after inoculation, both following Pseudomonadota (34.9%) (Fig. 2d). At the genus level, *Sphingomonas*, *Sphingosinicella*, and *Rhodospirillum* were the most abundant taxa in rhizosphere soils across different groups during the flowering stage (Fig. 2e). Rhizoplane soils of the control group were dominated by *Pseudomonas* (13.8%), *Sphingomonas* (6.5%), and *Pedobacter* (3.4%), whereas *Serratia* (10.7%) became predominant over *Pseudomonas* (9.6%) and *Sphingomonas* (8.1%) after inoculation (Fig. 2f). During the maturation stage, rhizosphere soils of the control group mainly contained *Sphingomonas* (7.4%), *Pseudomonas* (2.5%), and *Sphingosinicella* (2.4%), whereas *Rhodospirillum* (1.3%) replaced the third-ranked genus after inoculation (Fig. 2g). In rhizoplane soils of both groups, the top three genera were *Sphingomonas*, *Neobacillus*, and *Bacillus* at the maturation stage (Fig. 2h).

**Fig. 3:**
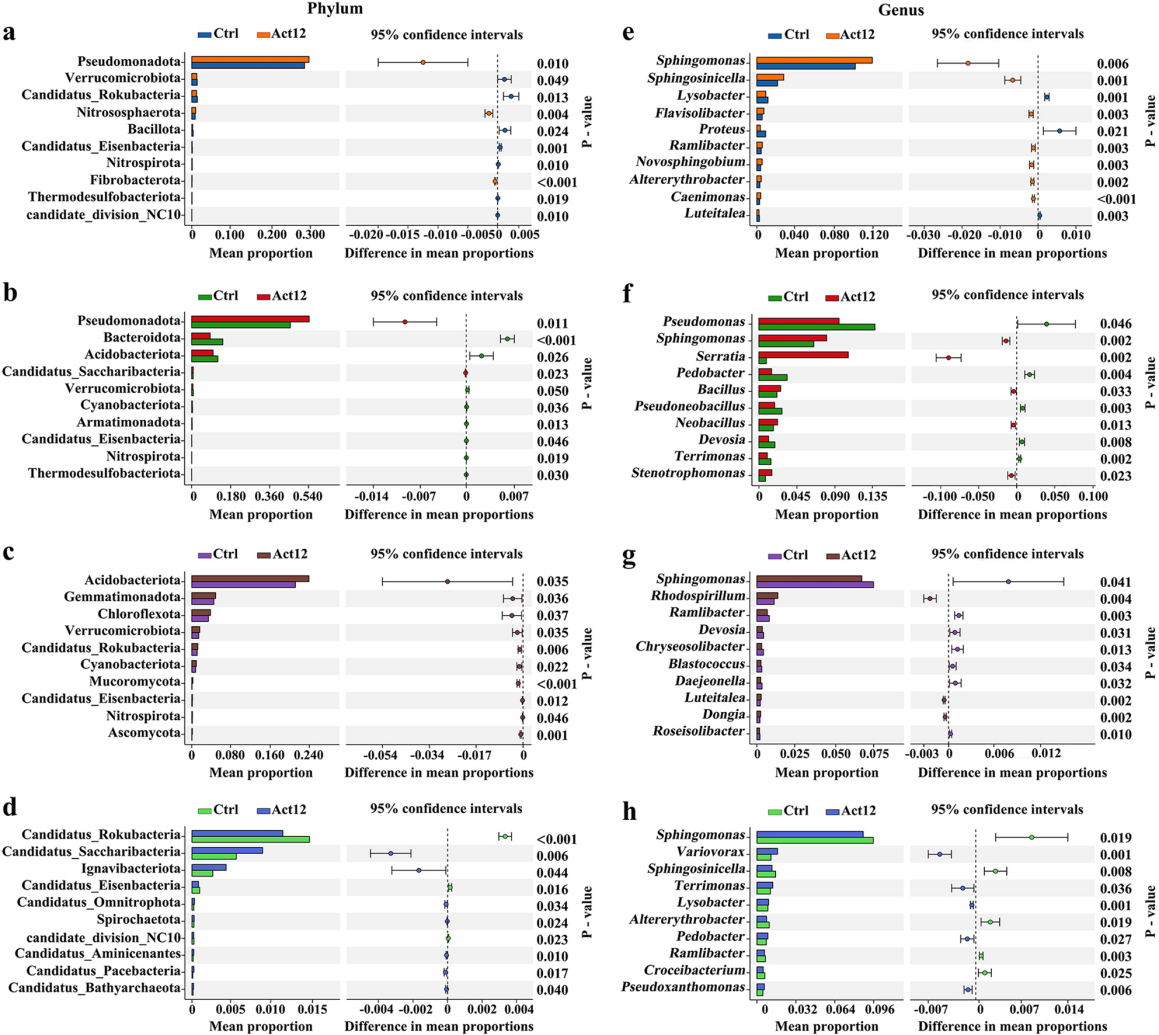
Relative abundance of the top 10 differential taxa. **a**, **e** rhizosphere at flowering; **b**, **f** rhizoplane at flowering; **c**, **g** rhizosphere at maturation; **d**, **h** rhizoplane at maturation

Among the differentially abundant phyla, microbial inoculation increased the relative abundances of Fibrobacterota, Nitrososphaerota, and Pseudomonadota in rhizosphere soils (by 3.9–49.3%) at the flowering stage. This was accompanied by decrease of Bacillota and Candidatus_Eisenbacteria (explicitly unlisted taxa among the top 10 differentially abundant taxa; by 4.9–31.0%) compared to the control group (Fig. 3a). Microbial inoculation also led to rhizoplane recruitment of Pseudomonadota (by 19.1%) and Candidatus_Saccharibacteria (by 10.8%), alongside depletion of Bacteroidota and andidatus_Eisenbacteria, (by 14.6–40.7%) (Fig. 3b). During the maturation stage, microbial inoculation promoted the occurrence of Mucoromycota and Ascomycota in rhizosphere soils (by 8.4–319.7%) (Fig. 3c). A similar effect was observed for Candidatus_Pacebacteria and Ignavibacteriota in rhizoplane soils (by 8.7–114.5%), where Candidatus_Rokubacteria, Candidatus_Eisenbacteria, and candidate_division_NC10 were suppressed (by 13.2–22.7%) (Fig. 3d). Furthermore, microbial inoculation resulted in higher relative abundances of *Altererythrobacter* (flowering) and *Rhodospirillum* (maturation) in rhizosphere soils, despite reducing *Proteus* (flowering) and *Chryseosolibacter* (maturation). In rhizoplane soils, microbial inoculation facilitated the occurrence of *Serratia* (flowering) and *Variovorax* (maturation), while suppressing *Devosia* (flowering) and *Croceibacterium* (maturation) (Fig. 3e–h).

Notably, microbial inoculation induced shifts in the phylum and genus that harbor the inoculant strain *S. pactum*. At the maturation stage, the relative abundance of Actinomycetota increased by 24.2% in rhizosphere soils and by 6.3% in rhizoplane soils after inoculation compared to the respective control group (Fig. S4a). The relative abundance of *Streptomyces* increased by 28.5% in inoculated rhizoplane soils at the same stage (Fig. S4b).

#### Metabolic pathway enrichment

**Fig. 4:**
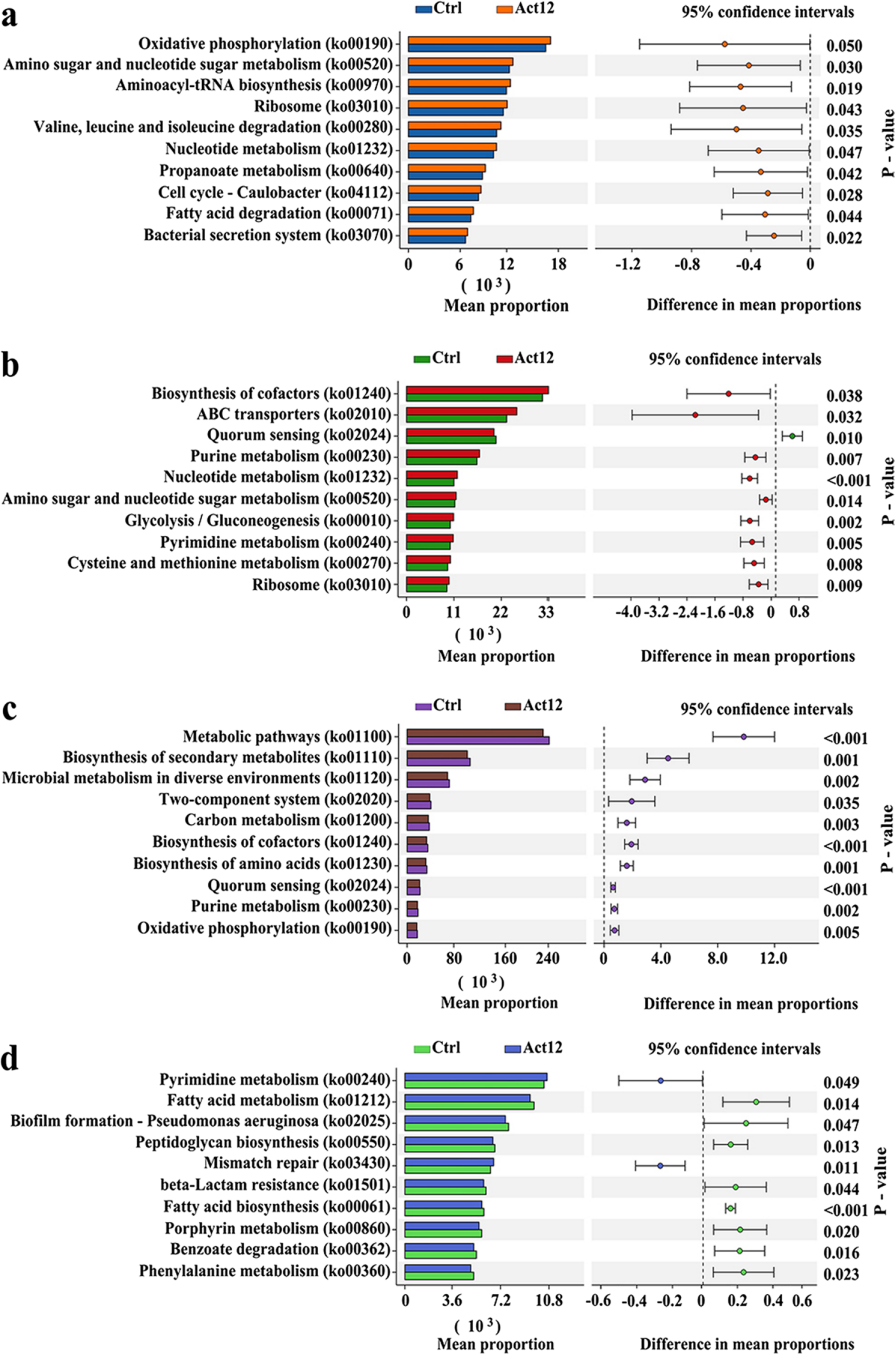
KEGG Level 3 functional annotation. **a** rhizosphere at flowering; **b** rhizoplane at flowering; **c** rhizosphere at maturation; **d** rhizoplane at maturation

KEGG Level 3 functional profiling of root-associated microbiomes revealed that at the flowering stage, microbial inoculation up-regulated oxidative phosphorylation, aminoacyl-tRNA biosynthesis, valine-leucine-isoleucine degradation, and fatty acid degradation pathways in rhizosphere soils (Fig. 4a). Additionally, ABC transporters, purine metabolism, pyrimidine metabolism, and cysteine and methionine metabolism pathways were up-regulated in rhizoplane soils after inoculation (Fig. 4b). At the maturation stage, microbial inoculation down-regulated the pathways related to biosynthesis of cofactors and two-component system in rhizosphere soils (Fig. 4c). In rhizoplane soils, pyrimidine metabolism and mismatch repair pathways were up-regulated after inoculation, with down-regulation of genes in phenylalanine metabolism pathway (Fig. 4d). Further, the top 10 differential metabolic pathways enriched in the top 10 differential genera were analyzed. There were consistent changes in the relative abundances of differential metabolic pathways and corresponding genera in response to microbial inoculation when compared to the respective control group (Fig. S5). This indicates that inoculation-induced changes in the taxa abundance of root-associated microbiomes potentially drove shifts in their metabolic functions.

### Characteristics of culturable microbiomes

#### Community composition

**Fig. 5:**
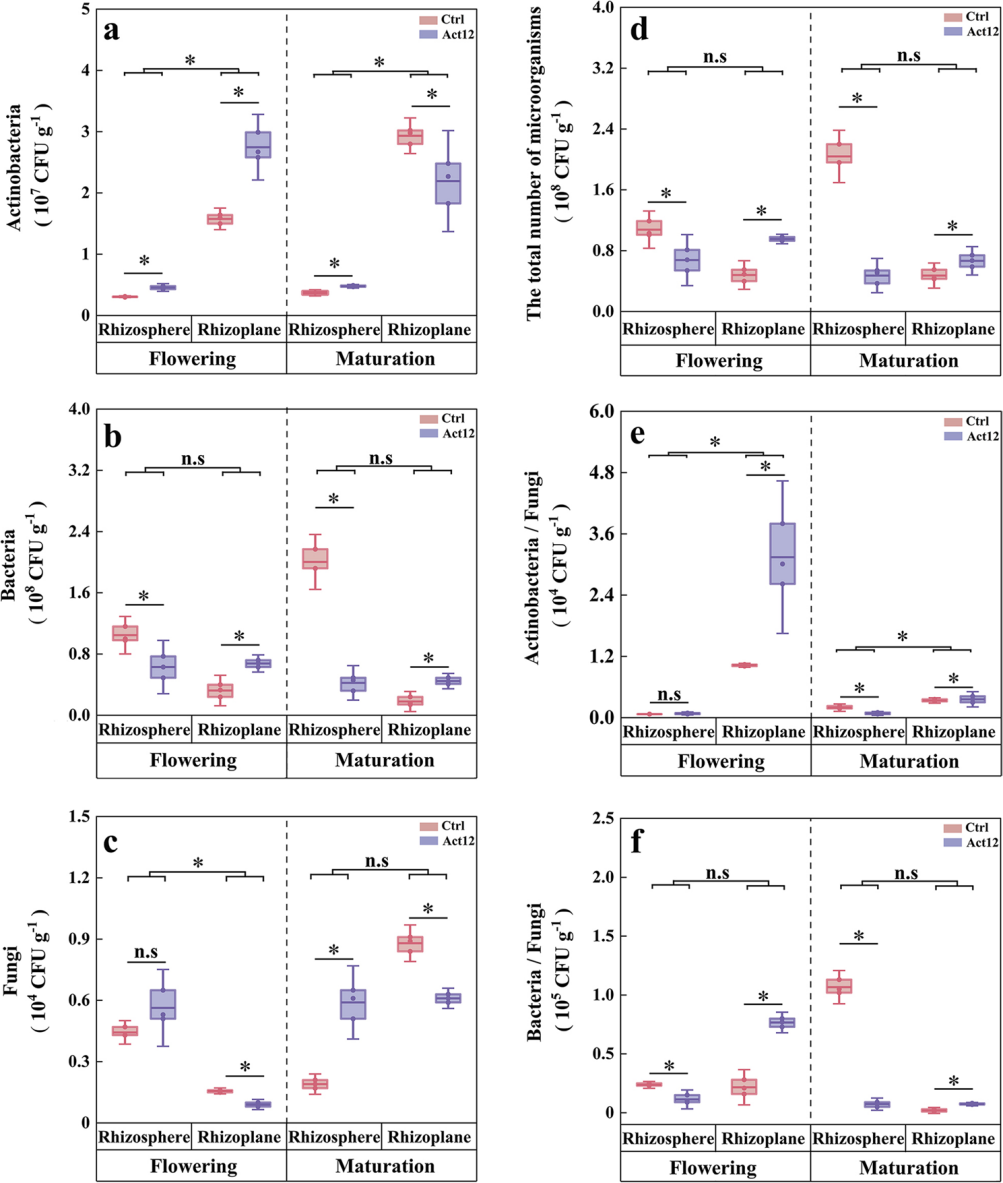
Culturable microbiome composition of in wheat root-associated soils following *S. pactum* Act12 inoculation. * *P* < 0.05

The culturable microbiomes showed distinct responses to microbial inoculation across soil compartments. Compared to the control group, the number of culturable actinobacteria in inoculated rhizosphere soils increased at the flowering (by 49.4%) and maturation (by 30.4%) stages. A greater increase of 74.4% was observed in the number of culturable actinobacteria in inoculated rhizoplane soils at the flowering stage, in contrast to a decrease of 25.2% at the maturation stage (Fig. 5a). Microbial inoculation reduced the number of culturable bacteria in rhizosphere soils at both stages by 39.8% and 78.8%, whereas the opposite pattern was observed in rhizoplane soils (Fig. 5b). Microbial inoculation additionally elevated the number of culturable fungi in rhizosphere soils at the maturation stage, with a reduced number in rhizoplane soils (Fig. 5c).

Rhizoplane soils showed higher number of culturable actinobacteria and A/F ratio than rhizosphere soils at both stages (Fig. 5a and e). In contrast, the number of culturable fungi in rhizoplane soils was lower than that in rhizosphere soils at the flowering stage (Fig. 5c). Microbial inoculation showed contrasting effects on the total number of culturable microorganisms, A/F ratio (except at flowering), and B/F ratio in rhizosphere soils (37.2–93.2% decrease) and rhizoplane soils (8.3–259.4% increase) (Fig. 5d–f). These findings suggest that microbial inoculation altered the community composition of culturable microbiomes by promoting selective recruitment of actinobacteria from the rhizosphere to the rhizoplane and exclusion of fungi from the rhizoplane.

#### Dominant strains

**Fig. 6:**
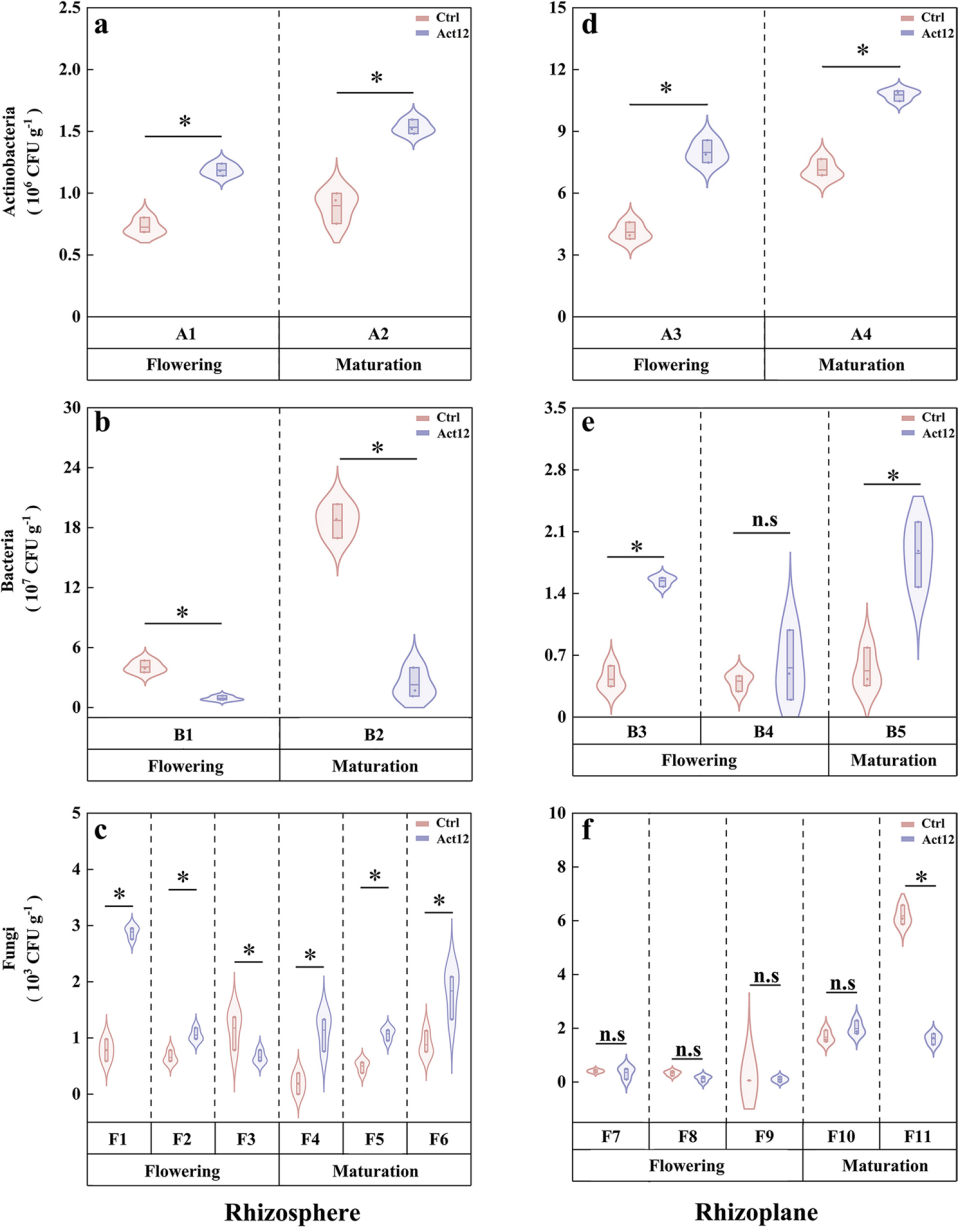
Numbers of dominant strains in wheat root-associated soils following *S. pactum* Act12 inoculation. * *P* < 0.05

A total of 20 dominant strains (4 actinobacteria, 5 bacteria, and 11 fungi) were isolated from rhizosphere and rhizoplane soils, and their molecular taxonomic characteristics are provided in Table S1. Compared to the control group, microbial inoculation increased the numbers of two actinobacterial strains (A1: *Kribbella italica*; A2: *Streptomyces seymenliensis*) in rhizosphere soils at the flowering (by 63.7%) and maturation (by 70.7%) stages, respectively. This corresponded to reduced numbers of one bacterial strain (B1: *Pseudomonas azotoformans*) at the flowering stage (by 77.3%) and another bacterial strain (B2: *Bacillus pumilus*) at the maturation stage (by 87.8%). Additionally, one fungal strain (F3: *Trichoderma harzianum*) decreased at the flowering stage after inoculation (by 44.2%). This contrasted with the increase of two fungal strains (F1: *Penicillium aurantiogriseum*; F2: *Penicillium janthinellum*) (by 268.2% and 60.6%). Another three fungal strains (F4: *Talaromyces purpureogenus*; F5: *Fusarium concolor*; F6: *Trichoderma atrobrunneum*) increased at the maturation stage after inoculation (by 108.9–505.1%) (Fig. 6a–c).

In rhizoplane soils, microbial inoculation increased the numbers of two actinobacterial strains (A3: *Kribbella italica*; A4: *Glycomyces lechevalierae*) and two bacterial strains (B3: *Microbacterium algeriense*; B5: *Advenella kashmirensis*) by 51.0–261.9% at specific stages compared to the control group. In contrast, the number of one fungal strain (F11: *Fusarium oxysporum*) was reduced by 74.0% at the maturation stage after inoculation (Fig. 6d–f).

### Functions of key strains

**Fig. 7:**
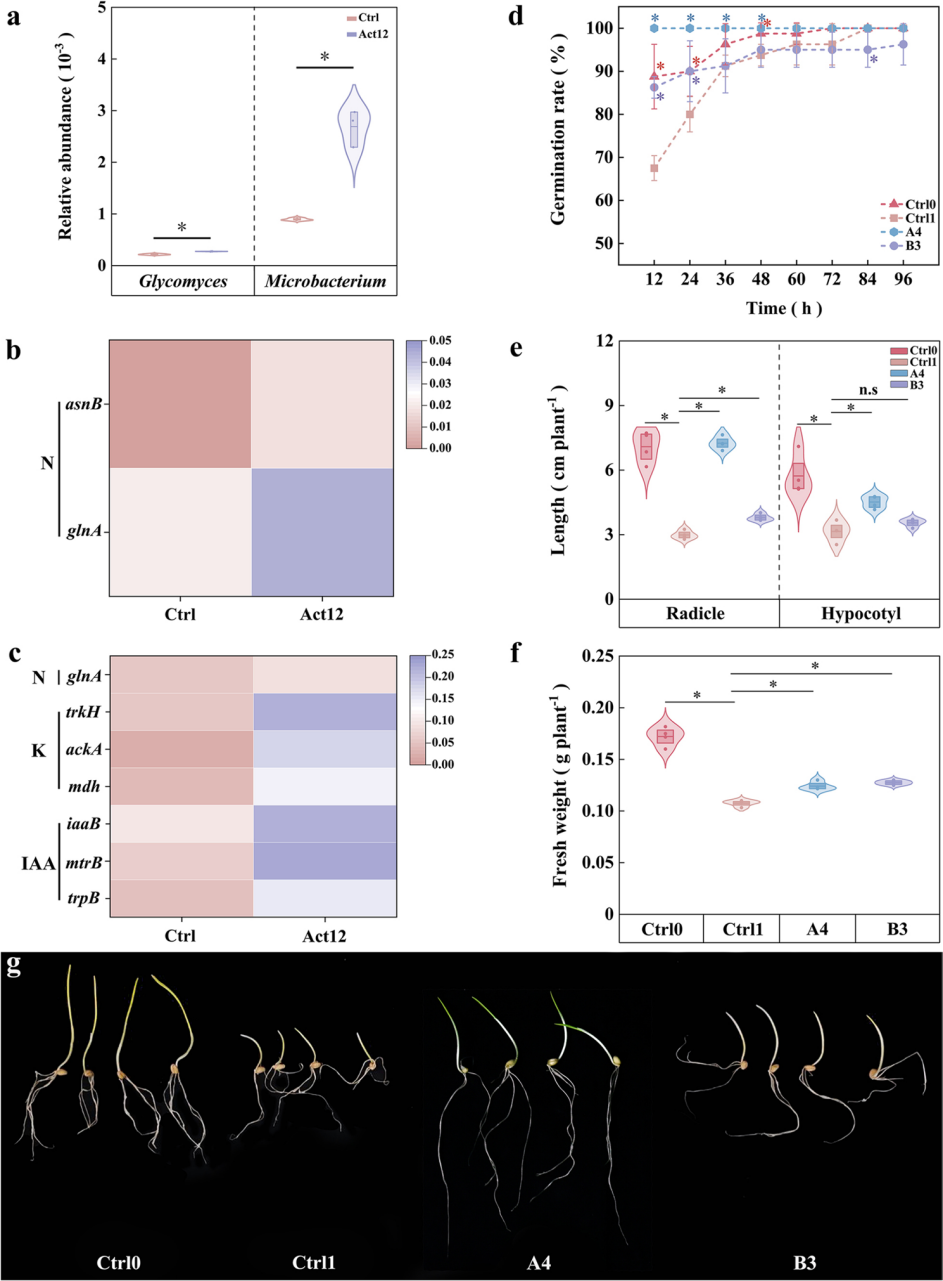
Abundances of key taxa (**a**) and relevant functional genes (**b**–**c**), and drought resistance mitigation in wheat seedlings following inoculation with key strains (**d**–**g**). *asnB*, *glnA* nitrogen fixation-related genes; *trk*H, *ack*A, *mdh* potassium solubilization-related genes; *trp*B, *mtr*B, *iaa*B IAA biosynthesis-related genes; Ctrl0 well-watered control; Ctrl1 15% PEG control; A4 *Glycomyces lechevalierae*; B3 *Microbacterium algeriense*. * *P* < 0.05

Integrating metagenomic profiles with culture-dependent results revealed that inoculation-induced shifts in the genus-level abundance of root-associated microbiomes mirrored changes in the numbers of dominant strains within the corresponding genera. For example, metagenomic analysis uncovered that microbial inoculation elevated the relative abundances of *Glycomyces* (by 25.9%) and *Microbacterium* (by 200.4%) in rhizoplane soils compared to the control group (Fig. 7a). This aligns with the pattern in the numbers of two key strains, *G. lechevalierae* A4 and *M. algeriense* B3 (Fig. 6d and e).

Functional analysis of the key strains A4 and B3 revealed their capabilities for nitrogen fixation, potassium solubilization, and IAA production (Table S2). KEGG-based annotation of nitrogen fixation, potassium solubilization, and IAA biosynthesis related genes was performed for *Glycomyces* (flowering) and *Microbacterium* (maturation) (Fig. 7b and c). Both *glnA* and *asnB* genes related to nitrogen fixation were identified in *Glycomyces*, and their abundances were elevated after inoculation compared to the control group (Fig. 7b). Microbial inoculation also up-regulated the abundance of *glnA* gene in *Microbacterium* (by 66.7%), in addition to multiple genes involved in potassium solubilization (*mdh*, *ackA*, and *trkH*, by 316.8–814.2%) and IAA biosynthesis (*mtrB*, *iaaB*, and *trpB*, by 130.4–246.1%) (Fig. 7c).

Compared to the normal control, PEG-induced drought stress inhibited seed germination and restricted seedling growth. In particular, seed germination rate was lowered by 23.9% at 12 h and by 11.1% at 24 h under drought stress (Fig. 7d), with 37.5–57.8% decrease in seedling radicle length, hypocotyl length, and fresh weight (Fig. 7e and f). Seed biopriming with strains A4 and B3 alleviated the effects of drought stress, as indicated by increased germination rates at 12 h (by 48.1% and 27.8%) and 24 h (by 25.0% and 12.5%) compared to the drought control. Seedling radicle length, hypocotyl length, and fresh weight were improved by 12.7–142.5% in the A4 and B3 inoculation treatments (Fig. 7d–f). These results support the functional role of *G. lechevalierae* A4 and *M. algeriense* B3 in alleviating drought-induced impairment of early wheat growth (Fig. 7d–g).

### Linkage between plant performance and root-associated microbiomes

**Fig. 8:**
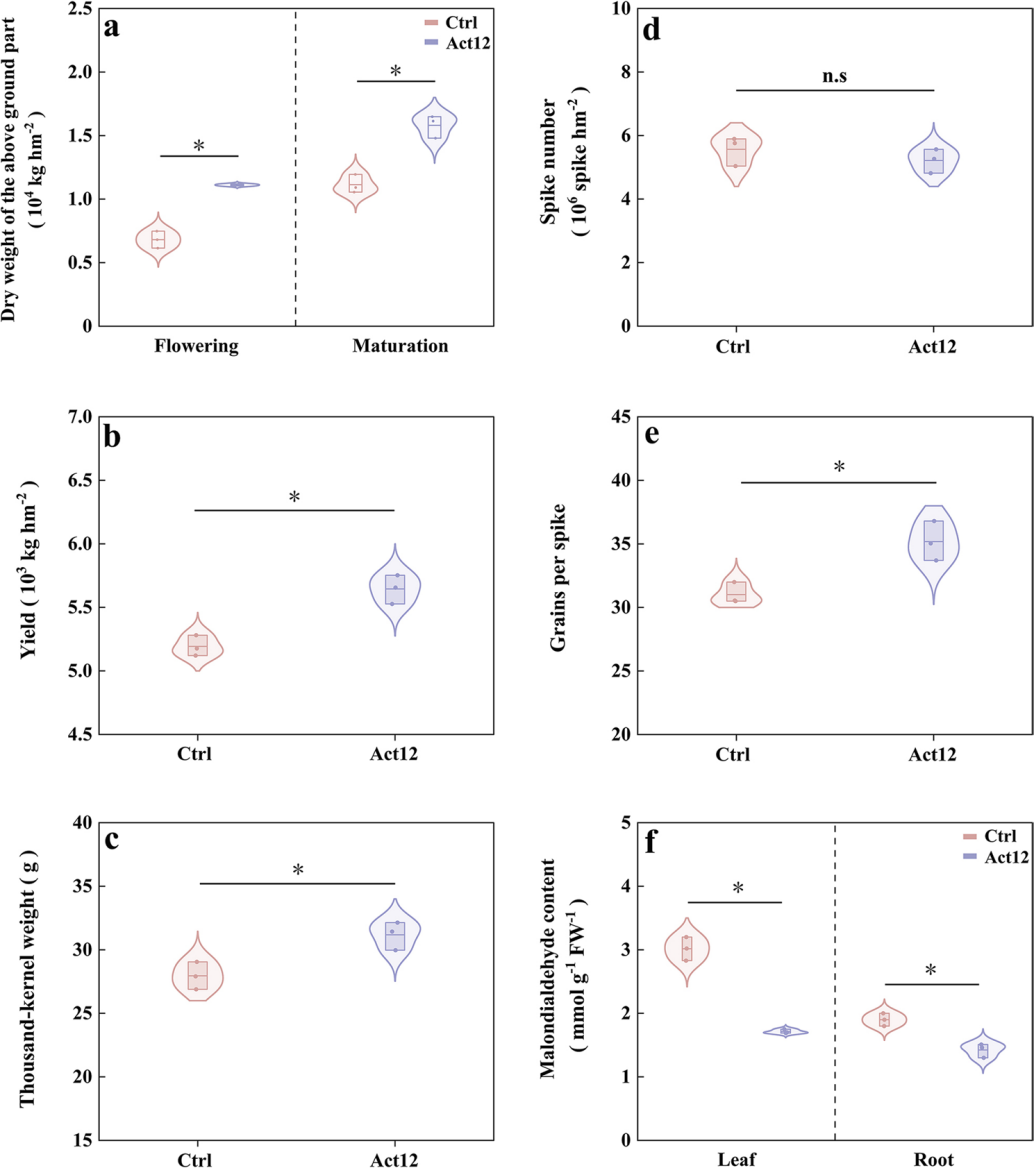
Wheat biomass, yield, yield components, and malondialdehyde content in wheat plants following *S. pactum* Act12 inoculation. * *P* < 0.05

Compared to the control group, microbial inoculation improved aboveground dry weight in wheat plants by 63.2% at flowering and by 41.9% at maturation (Fig. 8a), along with 8.7% increase in grain yield (Fig. 8b). Yield components responded differentially to inoculation, as exemplified by increase in thousand-kernel weight (by 11.6%) and grains per spike (by 13.4%), with marginal change in spike number (Fig. 8c–e). Furthermore, leaf and root MDA contents respectively decreased by 43.2% and 25.1% at the flowering stage after inoculation (Fig. 8f).

**Fig. 9:**
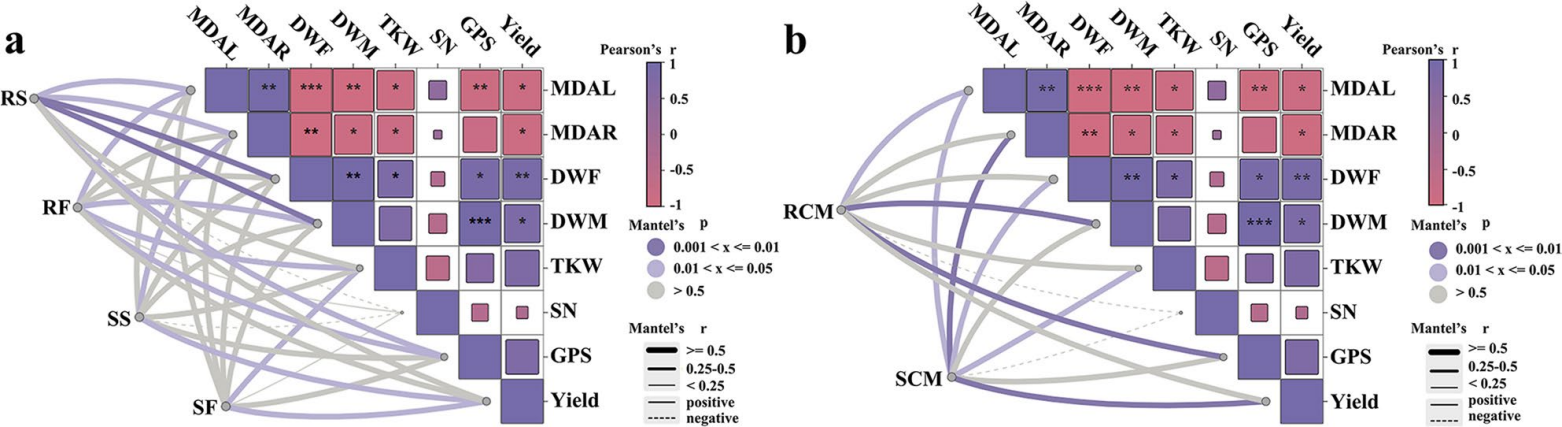
Mantel correlations between plant performance and root-associated microbial profiles derived from shotgun metagenome-based (**a**) and culture-dependent approaches (**b**). RS rhizosphere microbiome structure, RF rhizosphere microbiome function, SS rhizoplane microbiome structure, SF rhizoplane microbiome function, RCM rhizosphere culturable microbiome, SCM rhizoplane culturable microbiome, MDAL malondialdehyde content in wheat leaves, MDAR malondialdehyde content in wheat roots, DWF dry weight of the aboveground part at the flowering stage, DWM dry weight of the aboveground part at the maturation stage, TKW thousand-kernel weight, SN spike number, GPS grains per spike. * *P* < 0.05, ** *P* < 0.01, and *** *P* < 0.001

Pearson’s correlation analysis indicated that leaf and root MDA contents were negatively correlated with aboveground dry weight at both stages, thousand-kernel weight, and grain yield (*P* < 0.05 or *P* < 0.001; Fig. 9). Aboveground dry weight at both stages was positively correlated with grains per spike and grain yield (*P* < 0.05, *P* < 0.01, or *P* < 0.001). Mantel test revealed that regardless of the technique used (shotgun metagenome sequencing or culture-dependent methods), rhizosphere microbiome structure and function were positively correlated with aboveground dry weight at maturation and grains per spike (*P* < 0.05 or *P* < 0.01). Rhizoplane microbiome structure and function were positively associated with grain yield (*P* < 0.05 or *P* < 0.01). The cross-method concordance strengthened the reported relationships between plant performance and root-associated microbiomes.

Discrepancies also emerged between metagenomic profiles (Fig. 9a) and culture-dependent results (Fig. 9b). Metagenomic analysis demonstrated that rhizosphere microbiome structure was correlated with leaf and root MDA contents and aboveground dry weight at flowering (*P* < 0.05 or *P* < 0.01). Rhizoplane microbiome function was positively associated with leaf MDA content and thousand-kernel weight (*P* < 0.05). Rhizoplane microbiome structure and function were respectively linked to aboveground dry weight at maturation and thousand-kernel weight (*P* < 0.05). Culture-dependent results indicated that rhizosphere culturable microbiome was positively correlated with leaf MDA contents (*P* < 0.05). Rhizoplane culturable microbiome was positively correlated with leaf and root MDA contents and aboveground dry weight at flowering (*P* < 0.05 or *P* < 0.001). These results suggest that unculturable and culturable microbiomes in rhizosphere and rhizoplane soils performed distinct functions in wheat performance and yield.

## Discussion

### Microbial inoculation enhances drought resistance in dryland winter wheat

Exogenous application of functional microorganisms provides a promising strategy for enhancing crop drought resistance and agricultural productivity [53]. Drought stress can cause lipid peroxidation in cell membranes, as indicated by MDA accumulation [54]. Under dryland conditions, seed biopriming with *S. pactum* Act12 resulted in lower MDA contents in wheat leaves and roots at the flowering stage, indicating improved drought resistance in plants. This change was coupled with an elevation in aboveground dry weight at both the flowering and maturation stages, contributing to higher grain yield in wheat crops. Further analysis of yield components indicated that inoculation of *S. pactum* Act12 effectively elevated thousand-kernel weight and grains per spike, mitigating drought stress in wheat yield.

Drought stress can accelerate the peak grain-filling rate, shorten the grain filling duration, and reduce the overall grain-filling efficiency, leading to a decrease in grain weight [55]. Additionally, drought stress is likely to exacerbate assimilate competition between spikes and stems, which diverts resources from reproductive tissues, accelerates floret degeneration, and reduces grains per spike [55, 56]. It has been reported that inoculation of exogenous arbuscular mycorrhizal fungi, *Bacillus amyloliquefaciens*, and *Funneliformis mosseae* enhances wheat and maize grain weight by accelerating grain-filling rates and facilitating photosynthate allocation to grains [12, 25, 57]. Taken together, facilitation of floral organ development, optimization of assimilate distribution, and acceleration of grain filling may explain grain yield improvement in dryland winter wheat mediated by exogenous *S. pactum* Act12.

### Microbial inoculation associated with structural and functional profiles of root-associated microbiomes

Root-associated microbiomes play a vital role in regulating host plant growth and health. Inoculation of exogenous functional microorganisms may enhance crop resistance to abiotic stresses by reshaping root-associated microbiomes [58, 59]. Metagenomic profiling revealed that while enhancing drought resistance in wheat, inoculation of *S. pactum* Act12 altered the structural profiles of both rhizosphere and rhizoplane microbiomes. This was exemplified by enrichment of *Altererythrobacter*, *Serratia*, and *Stenotrophomonas* at the flowering stage, and *Rhodospirillum* and *Variovorax* at the maturation stage. These taxa have the potential to alleviate drought-induced damage to crop plants [59–62].

Soil microorganisms perform specific metabolic functions to directly affect the physicochemical microenvironment, which in turn influences crop performance [63]. Inoculation of *S. pactum* Act12 stimulated wheat growth while reshaping the functional profiles of rhizosphere and rhizoplane microbiomes. For example, the pathways of oxidative phosphorylation and ABC transporters were up-regulated at the flowering stage, followed by enhancement of pyrimidine metabolism at the maturation stage. Oxidative phosphorylation provides energy for critical soil reactions, conferring plant resistance to drought stress [22, 64]. Up-regulation of ABC transporters facilitates microbial uptake of extracellular compounds, which may strengthen plant drought resistance by exchanging metabolic products between microorganisms and plants [65, 66]. The enhancement of pyrimidine metabolism in the soil may selectively recruit microorganisms that can improve plant drought resistance [22, 66]. Thus, manipulating root-associated microbiomes through selective enrichment of key taxa and functional pathways is a possible mechanism that allows *S. pactum* Act12 to enhance drought resistance in dryland winter wheat.

Notably, several functional categories were enhanced in inoculated soils at the flowering stage followed by a decline at the maturation stage, when compared to the control group. The flowering stage represents a pivotal developmental transition in plants, characterized by a shift from vegetative to reproductive growth. This stage features substantial nutrient and energy allocation, with elevated root exudation into the soil. Such physiochemical changes can trigger enhanced metabolic activity in root-associated microbiomes, promoting microbial functions in key ecological processes [67, 68]. As mature wheat plants stop growing, the roots exhibit sharply decreased metabolic activity and exudation of bioactive metabolites. Consequently, specific microbial populations are depleted within the root zone, leading to a loss of associated functional capacities [69]. These mechanisms may account for stage-specific variations in the structural and functional profiles of wheat root-associated microbiomes in response to exogenous *S. pactum* Act12.

### Rhizosphere and rhizoplane microbiomes respond divergently to microbial inoculation

The functions of root-associated microbiomes are intimately linked to the ecological niches established by their host plants [70]. Inoculation of *S. pactum* Act12 enhanced plant performance in dryland winter wheat, with different microbial taxa enriched in rhizosphere soils (e.g., *Altererythrobacter*) and rhizoplane soils (e.g., *Bacillus*, *Variovorax*). These enriched taxa have been reported to benefit plant growth [71]. The differential responses of rhizosphere and rhizoplane microbiomes to exogenous *S. pactum* Act12 can be attributable to various spatial ranges of soil compartments based on their radial distance from the root axis [72]. Root-derived metabolic products serve as the major carbon source for rhizosphere and the rhizoplane microorganisms, thus driving microbial activity and proliferation. Root exudates also regulate the root-zone microenvironment, indirectly shaping rhizosphere and the rhizoplane microbiomes [73–75]. Compared to their rhizoplane counterparts, rhizosphere microorganisms reside at greater distances from the roots and consequently exhibit diminished responsiveness to root exudates [24, 76].

Furthermore, inoculation of *S. pactum* Act12 led to up-regulation of oxidative phosphorylation (a pathway related to phosphorus availability) [77] and amino sugar and nucleotide sugar metabolism (pathways involved in soil nutrient cycling) [77] in rhizosphere soils. In rhizoplane soils, ABC transporters and cysteine and methionine metabolism (two pathways directly linked to IAA biosynthesis) [21] were up-regulated after inoculation. The contrasting functional responses of rhizosphere and rhizoplane microbiomes to exogenous *S. pactum* Act12 are presumably governed by their spatial separation from the root surface. Rhizosphere microorganisms located farther from the roots benefit plant nutrient uptake and root growth mainly through phosphorus solubilization and potassium mobilization [24, 78, 79]. Rhizoplane microorganisms modulate plant development primarily by active production of signaling molecules (e.g., pyocyanin), hormones (e.g., IAA), and enzymes (e.g., ACC deaminase) [80–82]. Collectively, our findings indicate that exogenous *S. pactum* Act12 mediates compartment-specific alterations in root-associated microbiomes. The phylogenetic restructuring and functional reprogramming of rhizosphere and rhizoplane microbiomes synergize to boost wheat growth and yield under dryland conditions.

### Integrating high-throughput sequencing with culture-dependent techniques for functional validation

Integration of high-throughput sequencing with culture-dependent methods provides complementary strengths, unlocking new possibilities for soil microbiome investigation [63]. The results obtained using the two different approaches showed certain discrepancies, likely due to the fact that shotgun metagenome sequencing captures genetic information from both culturable and unculturable microorganisms, whereas culture-based analysis retrieves only a minor subset [83]. Two key strains, *G. lechevalierae* A4 and *M. algeriense* B3, were screened out from rhizoplane soils based on the results from metagenomic profiling coupled with culture-dependent analysis. Functional validation assays confirmed that inoculation of these two strains separately enhanced drought resistance in wheat seedlings. Both strains demonstrated functional capabilities for nitrogen fixation, potassium solubilization, and IAA biosynthesis. Notably, associated functional genes were up-regulated in *Glycomyces* (flowering) and *Microbacterium* (maturation) in rhizoplane soils after inoculation. This led us to posit that once colonizing the soil, *S. pactum* Act12 may mediate functional reprogramming of microbiomes in the root zone by selectively recruiting key taxa, which confers drought resistance in wheat. Further research should conduct co-culture or colonization assays to confirm the recruitment links and elucidate the interplay between exogenous *S. pactum* Act12 and indigenous key strains.

Importantly, culture-dependent analysis revealed that *S. pactum* inoculation consistently increased the number of culturable actinobacteria (rhizoplane) and evaluated the A/F ratio (rhizosphere) in soils across plant growth stages. This pattern may arise from the fact that actinobacteria are comparatively tolerant to drought, whereas fungi exhibit greater sensitivity to moisture deficits [84]. Inoculation of *S. pactum* Act12 likely improved the community composition of culturable microbiomes by dual mechanisms. One possible mechanism is selective enrichment of plant-beneficial bacteria (e.g., *A. kashmirensis*) [85], and the other is suppression of potential harmful fungi (e.g., *T. harzianum*, *F. oxysporum*) [86, 87]. The resulting communities were functionally streamlined toward drought mitigation and plant growth promotion. This provides additional evidence that targeted manipulation of root-associated microbiomes is an effective strategy for enhancing crop resilience in wheat under water-limited conditions [20, 88].

## Conclusions

**Fig. 10:**
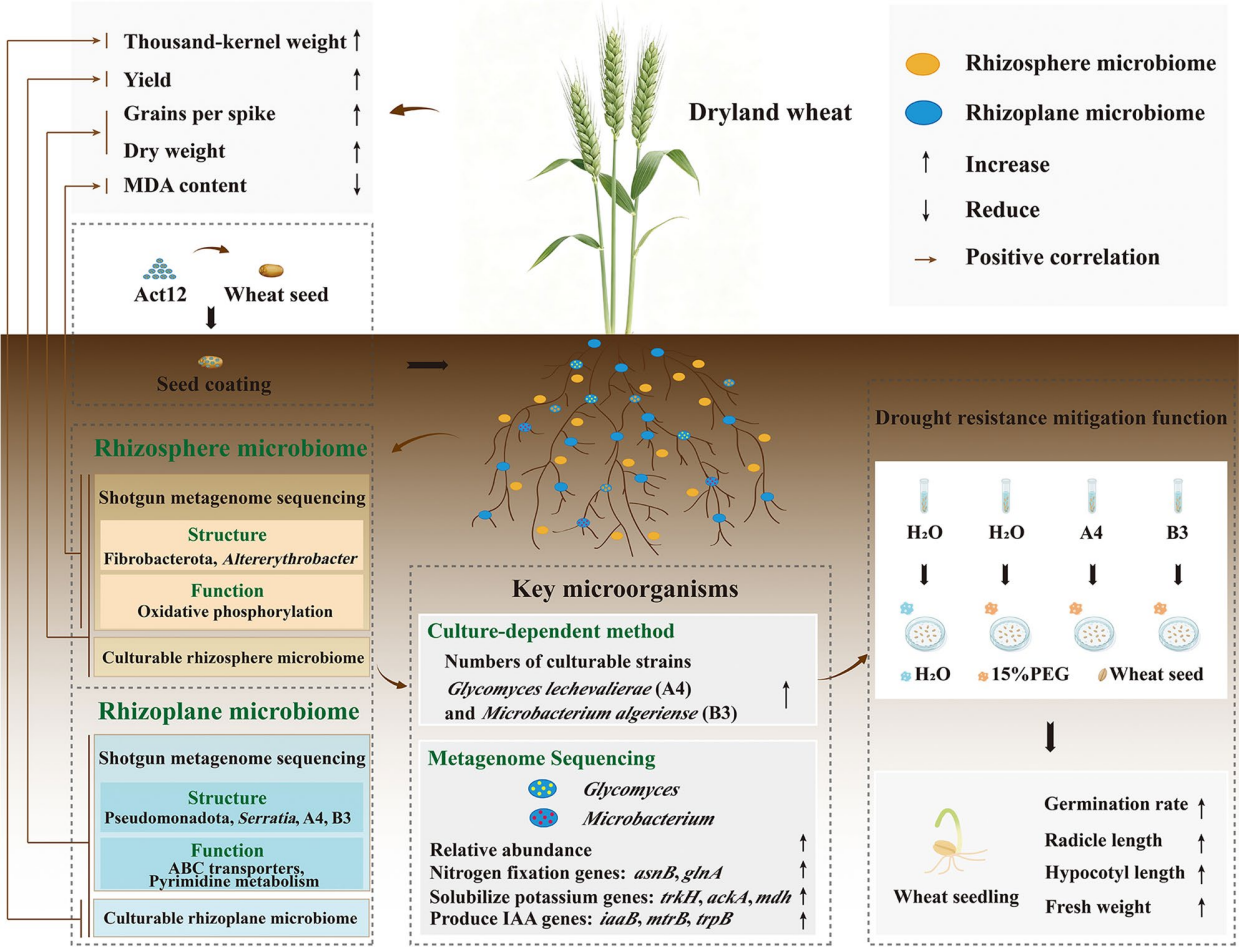
Proposed mechanisms for *S. pactum* Act12-mediated enhancement of drought resistance in dryland winter wheat by distinctively shaping rhizosphere and rhizoplane microbiomes

This study established a conceptual model depicting how exogenous *Streptomyces pactum* Act12 mediated enhancement of drought resistance in dryland winter wheat (Fig. 10). Going beyond previous studies that lacked spatial resolution, this study uncovered novel mechanisms for divergent modulation of rhizosphere and rhizoplane microbiomes by *S. pactum* Act12. Multi-dimensional evidence was obtained by integrating high-throughput sequencing and culture-dependent techniques. Seed biopriming with *S. pactum* Act12 improved wheat growth and grain yield under dryland conditions. This improvement in crop performance was accompanied by selective enrichment of drought-resistant taxa, such as Fibrobacterota and *Altererythrobacter* in rhizosphere soils. Pseudomonadota, *Serratia*, *Glycomyces lechevalierae* A4, and *Microbacterium algeriense* B3 were recruited in rhizoplane soils after inoculation. Microbial functional capacities were also up-regulated in inoculated soils, including pathways related to oxidative phosphorylation (rhizosphere) and ABC transporters and pyrimidine metabolism (rhizoplane). These findings broaden our understanding about the role of *Streptomyces* in crop resistance to drought, highlighting manipulation of root-associated microbiomes as a route to improve wheat yield.

## Supplementary Information

Supplementary Material 1

Supplementary Material 2

Supplementary Material 3

## Data Availability

The dataset analyzed during the current study is available from the China National Center for Bioinformation (CNCB, https://ngdc.cncb.ac.cn/gsub/) under BioProject number PRJCA049098.
