# Supplementary material for: Manipulating root-associated microbiomes to boost drought resistance in dryland winter wheat with Streptomyces pactum Act12
Source: BMC Microbiol. 2026 Feb 10;26:249. doi: 10.1186/s12866-026-04812-3 (PMC12990429; doi:10.1186/s12866-026-04812-3)
Supplement: Supplementary file 1 — Supplementary Material 1 [file 12866_2026_4812_MOESM1_ESM.docx]

**
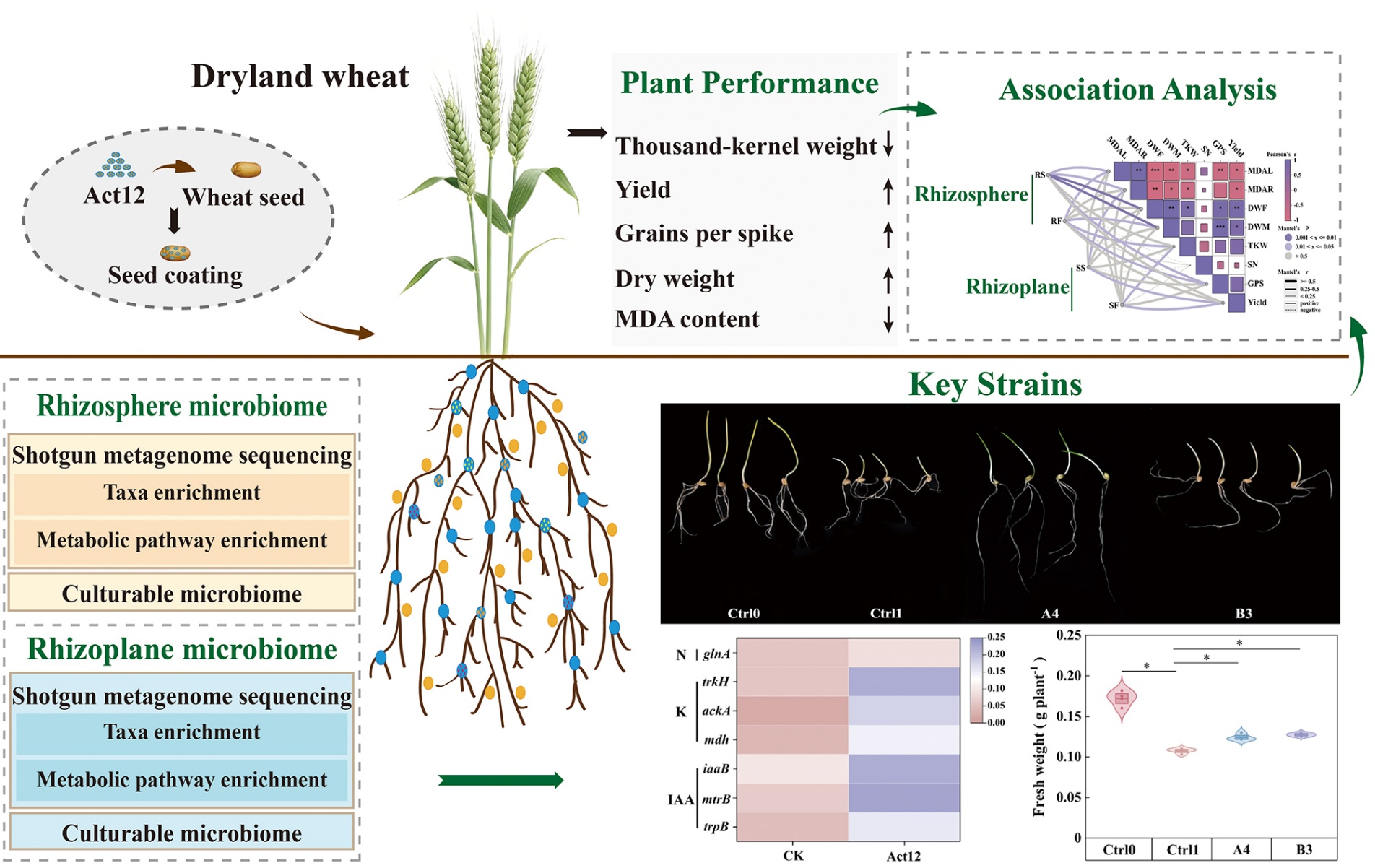
Graphical abstract** | *Streptomyces pactum* Act12 inoculation improved plant performance and grain yield for wheat cultivated under dryland conditions. Microbial inoculation distinctly modulated the structural and functional profiles of rhizosphere and rhizoplane microbiomes, consequently bolstering the crop’s drought resilience.
