# Supplementary material for: Manipulating root-associated microbiomes to boost drought resistance in dryland winter wheat with Streptomyces pactum Act12
Source: BMC Microbiol. 2026 Feb 10;26:249. doi: 10.1186/s12866-026-04812-3 (PMC12990429; doi:10.1186/s12866-026-04812-3)
Supplement: Supplementary file 2 — Supplementary Material 2 [file 12866_2026_4812_MOESM2_ESM.docx]

**Supplementary materials**

**Supplementary figure legends**

**Fig. S1** Precipitation and temperature conditions at the experimental site in Yuncheng, China.

**Fig. S2** Compartment-specific and Core-pan gene signatures of rhizosphere and rhizoplane soils under *Streptomyces pactum* Act12 inoculation. Act12R, rhizosphere of *S. pactum* Act12-inoculated wheat; Act12S, rhizoplane of *S. pactum* Act12-inoculated wheat; CtrlR, rhizosphere of non-inoculated wheat; CtrlS, rhizoplane of non-inoculated wheat.

**Fig. S3** Alpha diversity in root-associated soils affected by *S. pactum* Act12 inoculation. * *P* < 0.05.

**Fig. S4** Abundance changes of Actinomycetota and *Streptomyces* following *S. pactum* Act12 inoculation. * *P* < 0.05.

**Fig. S5** Relationships of top10 differential metabolic pathways enriched in the top10 differential microbial genera. **a** rhizosphere at flowering, **b** rhizoplane at flowering, **c** rhizosphere at maturation, **d** rhizoplane at maturation. Red / black labels denote significant increase / decrease in relative abundance following *S. pactum* Act12 inoculation versus the non-inoculated control. In the pathway panel, pink / blue indicate significant elevation / reduction of the genus-associated pathway; “-” indicates the pathway was not detected.

**Supplementary table legends**

**Table S1** Molecular identification of strains

**Table S2** Strains function analysis

**Supplementary figures**

**
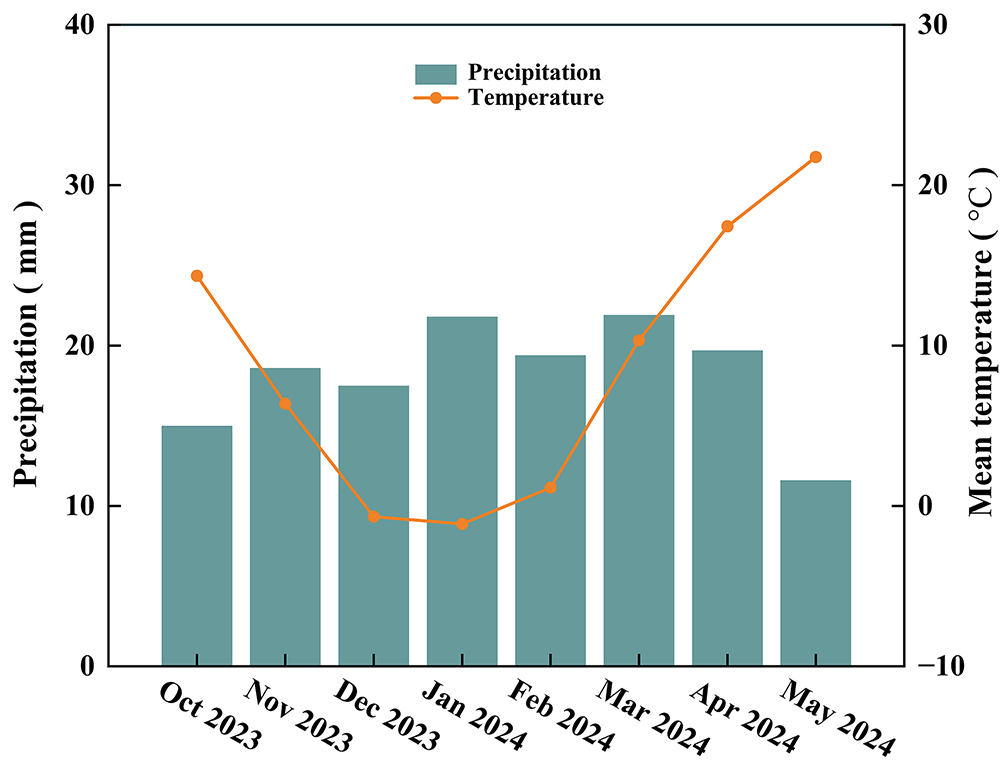
Fig. S1** Precipitation and temperature conditions at the experimental site in Yuncheng, China.


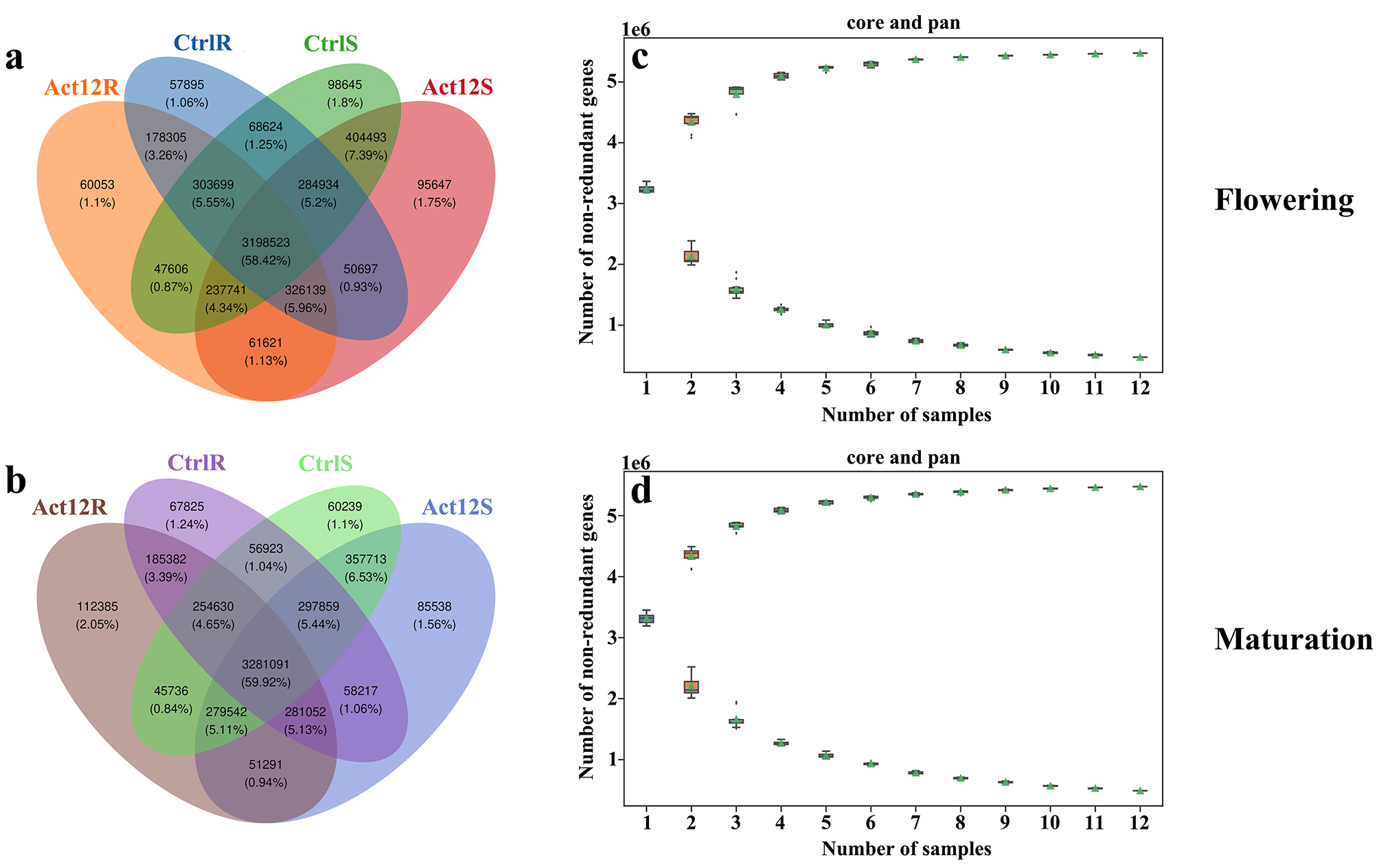


**Fig. S2** Compartment-specific and Core-pan gene signatures of rhizosphere and rhizoplane soils under *Streptomyces pactum* Act12 inoculation. Act12R, rhizosphere of *S. pactum* Act12-inoculated wheat; Act12S, rhizoplane of *S. pactum* Act12-inoculated wheat; CtrlR, rhizosphere of non-inoculated wheat; CtrlS, rhizoplane of non-inoculated wheat.

**
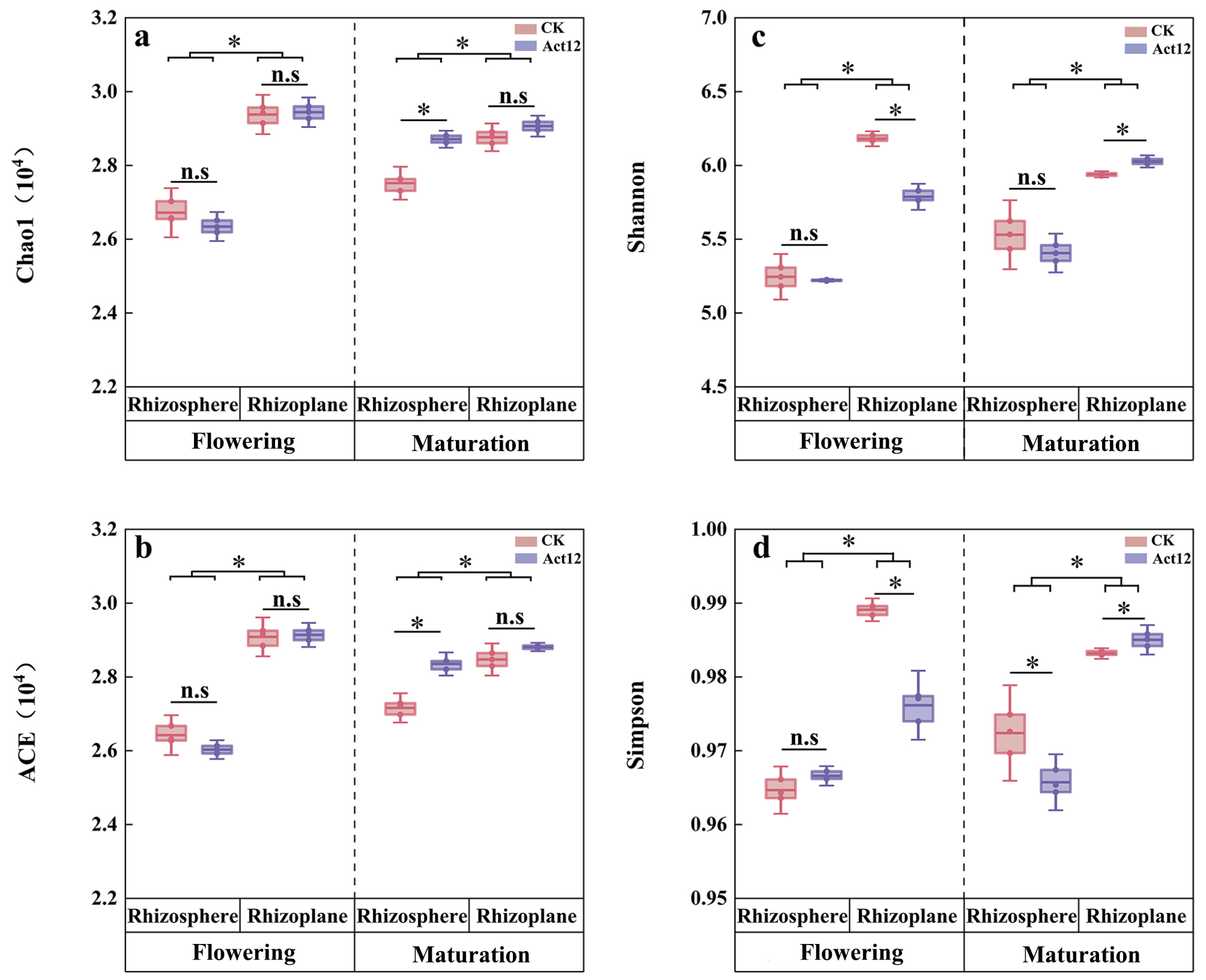
Fig. S3** Alpha diversity in root-associated soils affected by *S. pactum* Act12 inoculation. * *P* < 0.05.

**
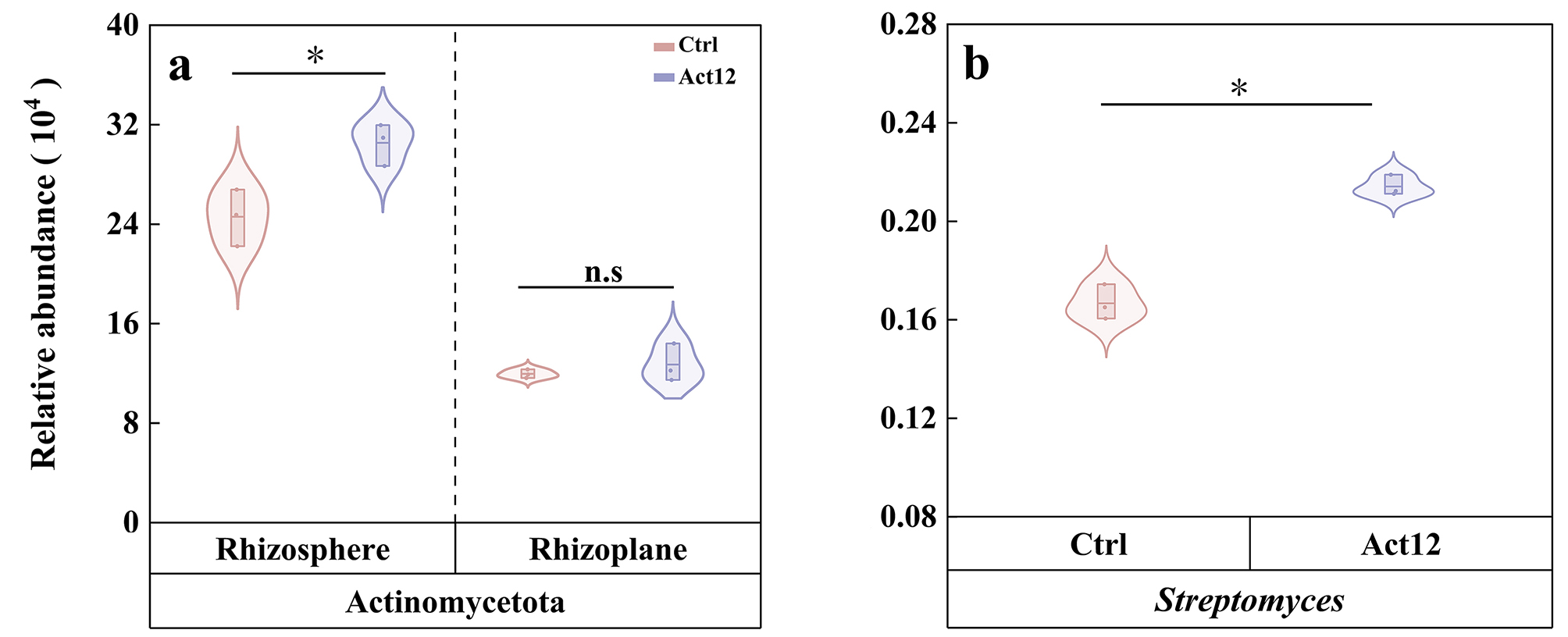
Fig. S4** Abundance changes of Actinomycetota and *Streptomyces* following *S. pactum* Act12 inoculation. * *P* < 0.05.


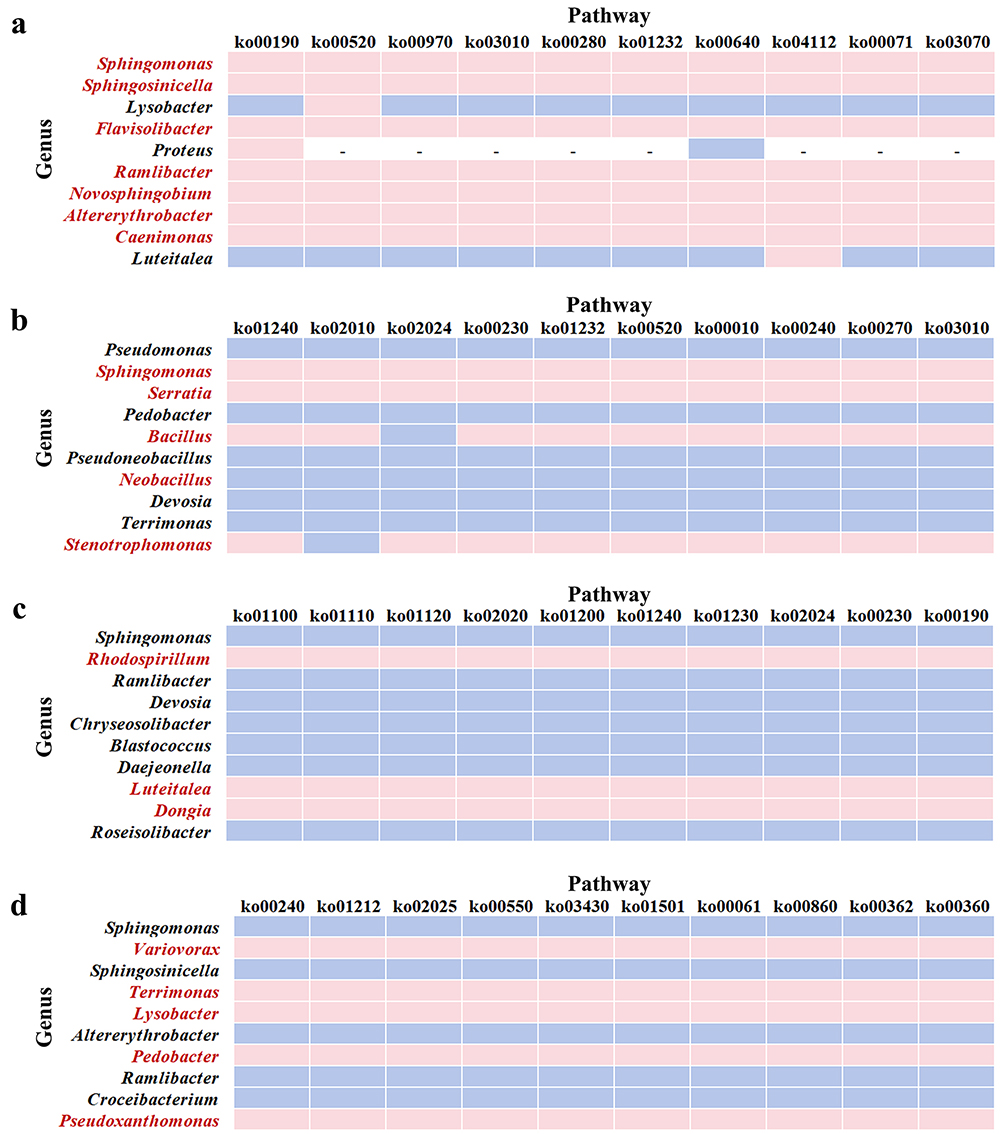


**Fig. S5** Relationships of top10 differential metabolic pathways enriched in the top10 differential microbial genera. **a** rhizosphere at flowering, **b** rhizoplane at flowering, **c** rhizosphere at maturation, **d** rhizoplane at maturation. Red / black labels denote significant increase / decrease in relative abundance following *S. pactum* Act12 inoculation versus the non-inoculated control. In the pathway panel, pink / blue indicate significant elevation / reduction of the genus-associated pathway; “-” indicates the pathway was not detected.

| **Supplementary tables**  **Table S1** Molecular identification of strains | | |
| --- | --- | --- |
| **Strains number** | **DNA identification results** | **Identities** |
| A1 | *Kribbella italica* | 99.01% |
| A2 | *Streptomyces seymenliensis* | 99.02% |
| A3 | *Kribbella italica* | 99.08% |
| A4 | *Glycomyces lechevalierae* | 98.87% |
| B1 | *Pseudomonas azotoformans* | 99.72% |
| B2 | *Bacillus pumilus* | 99.65% |
| B3 | *Microbacterium algeriense* | 99.30% |
| B4 | *Pseudomonas azotoformans* | 99.72% |
| B5 | *Advenella kashmirensis* | 99.86% |
| F1 | *Penicillium aurantiogriseum* | 99.10% |
| F2 | *Penicillium janthinellum* | 99.27% |
| F3 | *Trichoderma harzianum* | 99.31% |
| F4 | *Talaromyces purpureogenus* | 99.63% |
| F5 | *Fusarium concolor* | 99.22% |
| F6 | *Trichoderma atrobrunneum* | 99.49% |
| F7 | *Trichoderma peberdyi* | 99.48% |
| F8 | *Fusarium concolor* | 99.20% |
| F9 | *Penicillium cyclopium* | 99.81% |
| F10 | *Penicillium cellarum* | 99.64% |
| F11 | *Fusarium oxysporum* | 100.00% |

| **Table S2** Strains function analysis | | | | | | | | |
| --- | --- | --- | --- | --- | --- | --- | --- | --- |
| **Strain** | **Produce ACC deaminase** | **Dissolve organic phosphorus** | **Dissolve inorganic phosphorus** | **Nitrogen fixation** | **Solubilize potassium** | **Siderophore production** | **Produce EPS** | **Produce IAA** |
| A4 | － | － | － | ＋ | ＋ | － | － | ＋ |
| B3 | － | － | － | ＋ | ＋ | － | － | ＋ |
| Note: "+" indicates the presence of this function, while "-" indicates the absence of it. | | | | | | | | |
