# Supplementary material for: Manipulating root-associated microbiomes to boost drought resistance in dryland winter wheat with Streptomyces pactum Act12
Source: BMC Microbiol. 2026 Feb 10;26:249. doi: 10.1186/s12866-026-04812-3 (PMC12990429; doi:10.1186/s12866-026-04812-3)
Supplement: Supplementary file 3 — Supplementary Material 3 [file 12866_2026_4812_MOESM3_ESM.docx]

**Highlights**

- *Streptomyces pactum* inoculation enhanced wheat performance in dryland agroecosystem.
- Metagenomic and culture-dependent methods accelerates key microbial strain screening.
- Microbial inoculation differentially altered rhizosphere and rhizoplane microbiomes.
